# Intrinsic group behaviour: Dependence of pedestrian dyad dynamics on principal social and personal features

**DOI:** 10.1371/journal.pone.0187253

**Published:** 2017-11-02

**Authors:** Francesco Zanlungo, Zeynep Yücel, Dražen Brščić, Takayuki Kanda, Norihiro Hagita

**Affiliations:** 1 ATR International, Kyoto, Japan; 2 Okayama University, Okayama, Japan; 3 Faculty of Engineering, University of Rijeka, Rijeka, Croatia; University of Cologne, GERMANY

## Abstract

Being determined by human social behaviour, pedestrian group dynamics may depend on “intrinsic properties” such as the purpose of the pedestrians, their personal relation, gender, age, and body size. In this work we investigate the dynamical properties of pedestrian dyads (distance, spatial formation and velocity) by analysing a large data set of automatically tracked pedestrian trajectories in an unconstrained “ecological” setting (a shopping mall), whose apparent physical and social group properties have been analysed by three different human coders. We observed that females walk slower and closer than males, that workers walk faster, at a larger distance and more abreast than leisure oriented people, and that inter-group relation has a strong effect on group structure, with couples walking very close and abreast, colleagues walking at a larger distance, and friends walking more abreast than family members. Pedestrian height (obtained automatically through our tracking system) influences velocity and abreast distance, both growing functions of the average group height. Results regarding pedestrian age show that elderly people walk slowly, while active age adults walk at the maximum velocity. Groups with children have a strong tendency to walk in a non-abreast formation, with a large distance (despite a low abreast distance). A cross-analysis of the interplay between these intrinsic features, taking in account also the effect of an “extrinsic property” such as crowd density, confirms these major results but reveals also a richer structure. An interesting and unexpected result, for example, is that the velocity of groups with children *increases* with density, at least in the low-medium density range found under normal conditions in shopping malls. Children also appear to behave differently according to the gender of the parent.

## Introduction

An urban “physical” crowd [1, 2], i.e. a large number of people that share the same location, but not necessarily a social identity (i.e., they are not a “psychological crowd”), is nevertheless characterised by the presence of a large number of social groups, i.e. groups of people that know each other and move together, possibly while socially interacting. The ratio between individual pedestrians and pedestrians moving in groups may change considerably between different environments and at different times of the day [3–5], but it is in general never negligible, with groups representing up to 85% of the walking population [6, 7]. Despite this empirical evidence about the importance of groups, the standard approach in microscopic (agent-based) pedestrian modelling has been for long time to assume that the crowd is composed of individuals, moving without any preferential ties to other pedestrians. This is an extremely strong simplification of the system, although it was obviously understandable as a first approach to the problem. Nevertheless, it is intuitive that groups behave in a specific way (they move together and close) and their presence should clearly influence the dynamics of the crowd, and not taking in account the group component of crowds may have an impact on the planning of buildings and emergency evacuation plans. Indeed, [8] suggests that “the typical response to a variety of threats and disasters is not to flee but to seek the proximity of familiar persons and places”. On the other hand, [9] shows how proxemic (group) behaviour may strongly influence macroscopic crowd dynamics.

As for a critical case where omitting the existence of groups in pedestrian crowds may have a severe effect on public safety, we may consider the evacuation process during the 2011 Tohoku great earthquake and the following tsunami. It has been reported [10] that around 48% of people that evacuated by foot the city of Sendai (Miyagi, Japan) did it by moving in groups, with a probable effect on evacuation times (for the smaller city of Kamaishi, in Iwate prefecture, the figure was as high as 71%).

One of the advantages of a “microscopic” approach to crowd dynamics, i.e. an approach that includes a description of individual pedestrians, as opposed to an approach describing only macroscopic observables such as crowd density [11], is the possibility to simulate individual differences, psychological aspects and social interactions [12, 13]. Nevertheless, for this approach to be possible, a detailed and quantitative understanding of such differences is needed.

Indeed, in recent years, a few studies concerning empirical observations and mathematical modelling of the groups’ characteristic configuration and velocity have been introduced [3, 6, 7, 14–32]. As an example, while many authors describe group motion just as a tendency to move in proximity, [7] introduced a tendency to walk aligned in order to facilitate social interaction. Following their example, [21] assumed that groups move in an abreast formation while unconstrained, but assume a *V* formation at larger densities or in narrower environments, and walk in a line in even more congested situations. In a recent series of papers [3, 4, 33], we focused on the development of a mathematical model to describe group interaction, and specifically the group spatial structure and velocity. The model proposed in [3] introduced a non Newtonian [34] potential for group interaction on the basis of few and intuitive ideas about social interaction in pedestrian groups, and its predictions for group size, structure and velocity are in agreement with the observed natural behaviour of pedestrians. In [4] we studied a large data set of pedestrian trajectories to see how an *extrinsic*, i.e., environmental, property such as crowd density influences the dynamics of groups, and in [33] we introduced a mathematical model to explain such a crowd density effect on groups (density is probably only one of the environmental properties affecting group dynamics, a second one being the environment architectural features such as corridor width [35]).

Nevertheless, we may expect that a social behaviour such as walking in groups depends also on *intrinsic* properties of the groups. It is known by studies with subjects that age, gender and height affect walking speed [36], but here we are interested on how group behaviour is affected by the nature of the group itself: not only by the characteristics of the individuals that compose it, but also by the relation between them, which is expected to have a strong impact on group dynamics (see [14, 20, 37–41] and our preliminary study [42]). The work of [14] is particularly relevant for the current study. Although it was limited by being performed in a relatively narrow environment, and being based on lateral pictures that did not allow a quantitative analysis of spatial structure and velocity, it suggested gender-related differences in formation and velocity (males walking less abreast and at a faster speed than females, and mixed groups walking more abreast than same sex groups).

To study natural human behaviour we use a large *ecological* (i.e. obtained by observing unconstrained pedestrians in their natural environment, see [14]) data set to describe how the group spatial structure, size and velocity of *dyads* (two people groups) change based on the following intrinsic properties of groups:

1. **purpose** of movement,
2. **relation** between the members,
3. **gender** of the members,
4. **age** of the members,
5. **height** of the members.

According to [15], large groups are very unstable, and easily break up in sub-groups of two and three people, and thus only these smaller groups present a stable spatial formation. Furthermore, three people groups may be modelled using pair-wise interaction according to [3]. We plan nevertheless to perform a similar study for triads in the future.

Being the data set based on unconstrained trajectories of unknown pedestrians, such features are necessarily (with the exclusion of pedestrian height, obtained automatically through our tracking system [43]) *apparent*, i.e. based on the judgement of human coders, and thus an analysis of their reliability is performed. Furthermore, being social behaviour cultural dependent, the results are probably influenced by the place in which data were collected (a shopping mall in Osaka, western Japan; refer to [44, 45] for an analysis on cultural effect of pedestrian behaviour). Nevertheless, they provide a useful insight on how these intrinsic features affect in a quantitative way the behaviour of dyads.

## Materials and methods

### Data set

The pedestrian group data set used for this work is based on the freely available set [46], introduced by [4]. This set is again based on a very large pedestrian trajectory set [5], collected in a ≈ 900 m^2^ area of the Asia and Pacific Trade Center (ATC), a multi-purpose building located in the Osaka (Japan) port area. For the purpose of this work, in order to avoid taking in consideration the effect of architectural features of the environment [35], such as its width, we use data only from the corridor area as defined in [4].

The trajectories have been automatically tracked using 3D range sensors and the algorithm introduced in [43], which provides, along with the pedestrian position on the plane, the height of their head, for more than 800 hours during a one year time span. At the same time, we video recorded the tracking area using 16 different cameras. A subset of the video recordings were used by a human coder to identify the pedestrian social groups reported in data set [46]. The pedestrians in this data set are all *socially interacting*, i.e. they were, on the basis of conversation and gaze clues, coded as not only moving together, but also performing some kind of social interaction [3, 4].

#### An ecological data set

The data set concerns the natural behaviour of pedestrians, i.e. the pedestrians were behaving in an unconstrained way, and observed in their natural environment. Data were collected with the consent of local authorities and building managers. Posters explaining that an experiment concerning pedestrian tracking was being hold were present in the environment. All data are used only for research purposes, and while anonymous data (e.g. pedestrian positions) are released and shared with the research community, personal data (e.g. video images) are never released to respect the privacy of recorded pedestrians.

Collecting data in the pedestrians’ natural environment obviously presents some technical problems and an overall lower quality in tracking data (higher tracking noise), but it is an approach with growing popularity [47, 48], that allows for removing possible influence on pedestrian behaviour due to performing experiments in laboratories, i.e. artificial environments, using selected subjects following the experimenters’ instructions. This is extremely important for this study, since we may hardly believe that social pedestrian group behaviour could be observed in such controlled laboratory experiments [14].

#### Group composition coding

In order to obtain the “ground truth” for the inter-group composition and social relation, we proceeded similarly to our previous works [3, 4], and asked three different human coders to observe the video recordings corresponding to the data set [46] and analyse the group composition, and in detail to code, when possible,

1. the apparent purpose of the group’s visit to the area (**work** or **leisure**),
2. the apparent gender of their members,
3. their apparent relation: **colleagues**, **friends**, **family** or **couples** (the Japanese term used, *koibito*, could be translated also as “lovers”, and suggests the idea of a relatively young, unmarried couple),
4. their apparent age (in decades, such as 0-9, 10-19, etc.)

While one coder examined data from five different days (three working days and two holidays), corresponding to 1168 different socially interacting dyads, the other two examined only one day (283 dyads). The coders are not specialised in pedestrian studies, are not aware of our mathematical models of pedestrian behaviour (one of the coders that analysed data from a single day is a non-technical member of our lab, but she did not take part in the development of mathematical modelling or quantitative data analysis), and did not have access to our quantitative measurements of position and velocity. They thus relied only on visual features such as clothing, gestures, behaviour and gazing [49–51] to identify the groups’ social roles and composition. (Distance and velocity are obviously features of the pedestrians’ behaviour, but the coders had access only to visual clues concerning these properties, and not to quantitative measurements. Furthermore, they were not given instructions such as “friends walk closer than colleagues” or similar. They were simply told to use the available visual clues to code the social roles and composition).

#### Coders’ agreement

The coding process is obviously strongly dependent on the subjective evaluation of the coder. Nevertheless, the 283 dyads examined by all coders may be used to examine their agreement, and provide thus some information about the reliability of their coding. To this end, we use in S4 Appendix two different approaches. On one hand, we use the standard approach used in social sciences of directly comparing the results of the coding, through statistical indicators such as Cohen’s and Fleiss’s kappa, or Krippendorf’s alpha. On the other hand, we also use an approach more rooted in the “hard” sciences, and treat the different codings as independent experiments, and quantitatively and quantitatively compare the findings.

#### Trajectories

While our tracking system provides us with pedestrian positions and velocities at time intervals *δt* in the order of tens of milliseconds, we average pedestrian positions over time intervals Δ*t* = 0.5 s, to reduce the effect of measurement noise and the influence of pedestrian gait. As a result, we obtain pedestrian positions at discrete times *k*, as

$$\begin{array}{c} x(k\Delta t)=(x(k\Delta t),y(k\Delta t),z(k\Delta t)), \end{array}$$ (1)

where *z* gives the height of the top of the pedestrian head, and define pedestrian velocities in 2D as

$$\begin{array}{c} v\left(k\Delta t\right)=[(x(k\Delta t)-x((k-1)\Delta t))/\Delta t,(y(k\Delta t)-y((k-1)\Delta t))/\Delta t]. \end{array}$$ (2)

Following [3, 4], only data points with both the average group velocity *V* (Eq 3) and all individual velocities *v*_*i*_ larger 0.5 m/s, and with all pedestrian positions falling inside a square with side 2.5 m centred on the group centre, were used (these thresholds were again based on our analysis of probability distribution functions of group positions in [3, 4] and pedestrian velocities in [5]). The pedestrian trajectories, and the corresponding coding of group and individual features, are available in S1 Data Set.

#### Pedestrian height

Pedestrian height measurement is obviously subject to oscillations (see [43]). A major problem with height tracking is that there are situations in which the head is hidden or poorly tracked, and the pedestrian height is wrongly assigned as the height of the shoulders. To avoid this problem, for each pedestrian we first compute the median height over the whole trajectory, and then define the pedestrian height as the average $\bar{z}$ of all *z* measurements above the median. As discussed in [43] and [5], tracking errors in which the tracking ID is misassigned to a different pedestrian, or, in the worst of cases, to an object, are possible, in particular in crowded environments. Such errors obviously affect the measurement of the pedestrian height, but since our pedestrians move in group, the large majority of these errors may be identified by noticing that the pedestrians corresponding to the IDs in the group are not moving together anymore. For this reason, when computing the median of *z* we use only data points for which the distance between pedestrians in the group was less than 4 meters. This is a conservative threshold justified by our findings in [3, 4] suggesting that interacting groups have extremely low probability of having a distance larger than 2 meters.

By being smaller and thus more easily occluded, the tracking of children is more difficult than the tracking of adults (for example, a couple of children resulted to have a height in the 160-170 cm range, due to a confusion between the parent and children position). Since our statistical analysis identified a few interesting results related to children, we visually analysed the video recordings to verify that this problem did not affect significantly our findings.

#### Density

As we report in [4], group velocity and spatial configuration depend on crowd density. In this paper we follow again our main analysis of [4] and compute pedestrian density with a good spatial resolution (more than a good time resolution) as time averages over 300 seconds in a *L* = 0.5 meters square area. More details, along with a discussion of possible density definitions, may be found in [4] (refer also to [52] and [53] for possible alternative definitions of density).

### Quantitative observables

**Fig 1:**
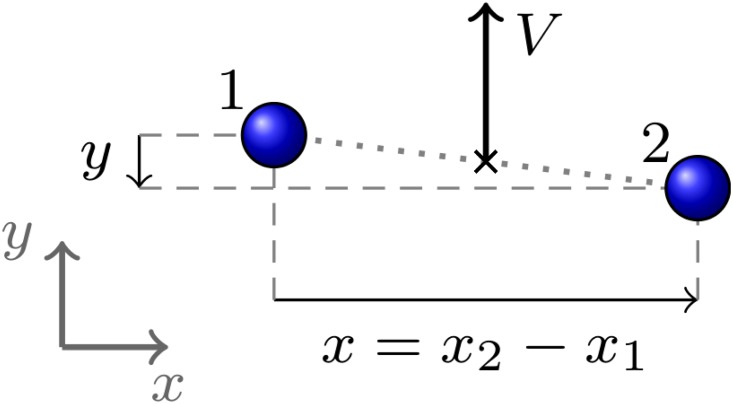
Group observables.

Based on our analysis performed in works [3, 4, 33], we define the following quantitative observables for the dynamics of a pedestrian dyad (Fig 1):

1. The *group velocity* *V*, defined as
  $$\begin{array}{c} V=|V|\;V=(v_{1}+v_{2})/2, \end{array}$$ (3)
  **v**_*i*_ being the velocities of the two pedestrians.
2. The *pedestrian distance* or *group size* *r*, defined as
  $$\begin{array}{c} r=|r|\;r=r_{1}-r_{2}, \end{array}$$ (4)
  **r**_*i*_ being the positions of the two pedestrians in the above reference frame.
3. The *group abreast distance* or *abreast extension* *x*, that may be defined as follows. First we identify a unit vector in the direction of **V**
  $$\begin{array}{c} \hat{g}=\frac{V}{V}. \end{array}$$ (5)
  For each pedestrian we compute the clockwise angle *θ*_*i*_ between $\hat{g}$ and **r**_*i*_, and define the projection of each **r**_*i*_ orthogonal to the velocity
  $$\begin{array}{c} x_{i}=r_{i}\sin\theta_{i}, \end{array}$$ (6)
  and define
  $$\begin{array}{c} x=|x_{2}-x_{1}|. \end{array}$$ (7)
4. We can also define the group extension in the direction of motion, or *group depth*, by
  $$\begin{array}{c} y_{i}=r_{i}\cos\theta_{i},\;y=\left|y_{2}-y_{1}\right|. \end{array}$$ (8)

As described in detail in S1 Appendix, to which the reader should refer for technical details, for each observable *O* and relation or composition category *k* we provide the number of groups $N_{g}^{k}$, the category average <*O*>_*k*_, standard deviation *σ*_*k*_ and standard error *ε*_*k*_, all based on the analysis of groups that contributed with at least 10 usable data points, and reported in tables as

$$\begin{array}{c} <O{>}_{k}\pm \varepsilon_{k}\;\;\left(\sigma \right). \end{array}$$ (9)

Furthermore we provide an analysis of the overall observable probability distribution function, and some parameters to estimate the differences between categories (ANOVA *p* values, effect size *δ*, coefficient of determination *R*^2^). The cross-analysis regarding the common effect of different “factors” (i.e., purpose, relation, gender, age and height) may be found in S3 Appendix.

## Results

### The effect of purpose

#### Overall statistical analysis

The results related to the purpose dependence of all observables concerning the 1088 dyads whose purpose was coded (and that provided enough data to be analysed) are shown in Table 1 (refer to S1 Appendix for an explanation of all terms). We have thus a very strong and significant evidence that pedestrians who visited the environment for working walk at a higher velocity and with a larger abreast distance, as shown by the comparison of averages and standard errors, and by the corresponding high *F* and *δ* and low *p* values (see S1 Appendix for definitions and meaning of these quantities). We have also a difference in distance and “group depth”, although its significance is less strong.

**Table 1:** Observable dependence on purpose for dyads. Lengths in millimetres, times in seconds.

Purpose	$N_{g}^{k}$	*V*	*r*	*x*	*y*
Leisure	716	1118 ± 7.3 (*σ* = 195)	815 ± 9.5 (*σ* = 253)	628 ± 6.1 (*σ* = 162)	383 ± 12 (*σ* = 334)
Work	372	1271 ± 8.2 (*σ* = 158)	845 ± 12 (*σ* = 228)	713 ± 8 (*σ* = 154)	332 ± 15 (*σ* = 289)
*F*_1,1086_		169	3.75	69.4	6.25
*p*		< 10^−8^	0.053	< 10^−8^	0.0126
*R*^2^		0.135	0.00344	0.0601	0.00572
*δ*		0.832	0.124	0.533	0.16

#### Probability distribution functions

**Fig 2:**
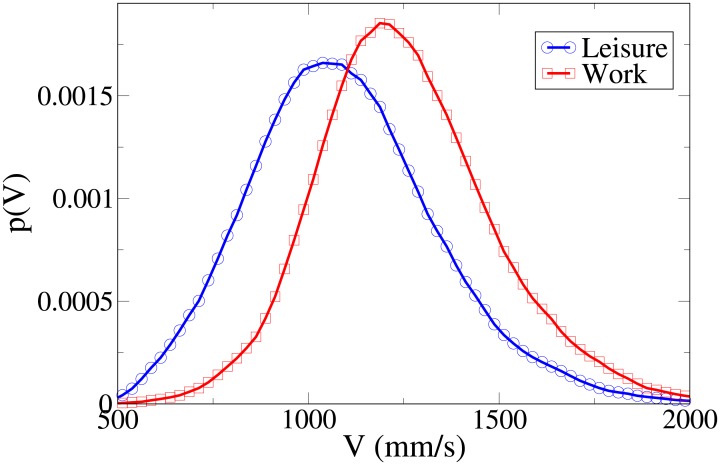
Pdf of the *V* observable according to purpose. Blue, centre of bin identified by circles: leisure oriented dyads. Red and squares: work oriented dyads. All pdfs in this work are shown after having been smoothed with a moving average filter.

**Fig 3:**
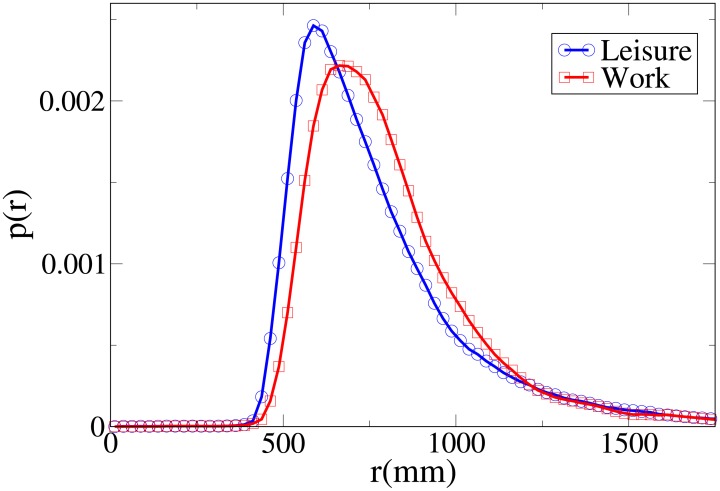
Pdf of the *r* observable according to purpose. Blue and circles: leisure oriented dyads. Red and squares: work oriented dyads.

**Fig 4:**
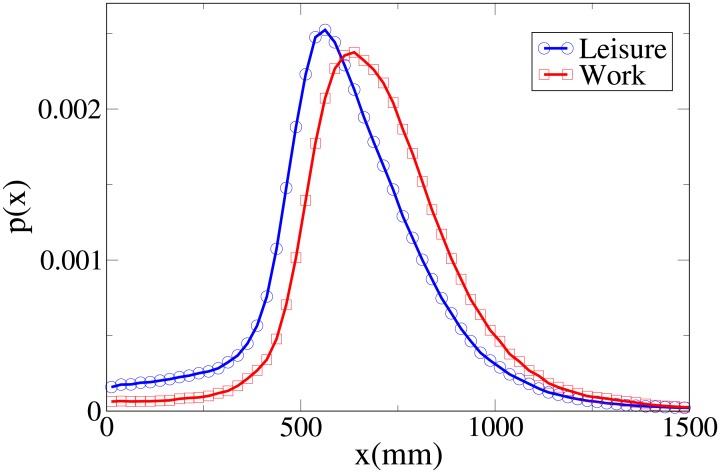
Pdf of the *x* observable according to purpose. Blue and circles: leisure oriented dyads. Red and squares: work oriented dyads.

**Fig 5:**
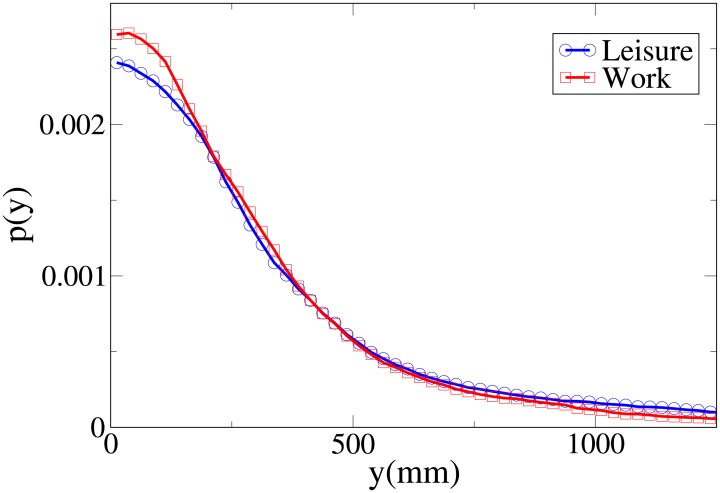
Pdf of the *y* observable according to purpose. Blue and circles: leisure oriented dyads. Red and squares: work oriented dyads.

We can get further insight about the differences in behaviour between workers and leisure oriented people by studying explicitly the probability distribution functions for the observables *V*, *r*, *x* and *y*, which are shown respectively in Figs 2, 3, 4 and 5, and whose statistical analysis is reported in S2 Appendix (refer again to S1 Appendix for the difference between the analysis reported in the main text an the one of S2 Appendix).

These pdfs provide an easy interpretation of the data. The *x* and *V* peaks and tails are displaced to higher values for workers. The *r* peak is also displaced to a higher value, but leisure distribution has a fatter tail. Correspondingly, the *y* distribution is slightly more spread in leisure oriented pedestrians. Furthermore, the *x* distribution presents a considerably higher value for low *x* values in the “leisure” case. These latter considerations show that while “workers” walk strongly abreast, the “leisure” dyads are less ordered.

#### Further analysis

In S3 Appendix we further analyse these results, to understand the effect on them of age, gender, density and height, while in S4 Appendix we verify that the major findings are confirmed by all coders. We may see that in general, even when age, gender, density and height are kept fixed, the results exposed above are confirmed by this further analysis.

### The effect of relation

Groups may also be analysed according to the relation between their members (colleagues, couples, friends, family). There is obviously a strong overlap between the “colleagues” category and the “work” one analysed above (and obviously between “leisure” and the three categories of couples, friends and families), but since they are conceptually different (colleagues could visit the shopping mall for lunch, or for shopping outside of working time), we will provide an independent analysis (although in the cross-analysis of S3 Appendix we usually drop the analysis of purpose and focus on relation).

#### Overall statistical analysis

The results related to the relation dependence for all observables concerning the 1018 dyads whose purpose was coded (and that provided enough data to be analysed) are shown in Table 2.

**Table 2:** Observable dependence on relation for dyads. Lengths in millimetres, times in seconds.

Relation	$N_{g}^{k}$	*V*	*r*	*x*	*y*
Colleagues	358	1274 ± 8.3 (*σ* = 157)	851 ± 12 (*σ* = 231)	718 ± 8.3 (*σ* = 157)	334 ± 15 (*σ* = 292)
Couples	96	1099 ± 17 (*σ* = 169)	714 ± 22 (*σ* = 219)	600 ± 15 (*σ* = 150)	291 ± 24 (*σ* = 231)
Families	246	1094 ± 13 (*σ* = 197)	863 ± 19 (*σ* = 302)	583 ± 11 (*σ* = 171)	498 ± 25 (*σ* = 391)
Friends	318	1138 ± 11 (*σ* = 200)	792 ± 11 (*σ* = 199)	662 ± 7.5 (*σ* = 134)	314 ± 15 (*σ* = 268)
*F*_3,1014_		60.7	12.2	42.3	21.4
*p*		< 10^−8^	7.39⋅10^−8^	< 10^−8^	< 10^−8^
*R*^2^		0.152	0.0349	0.111	0.0595
*δ*		1.03	0.529	0.828	0.587

We may see that, as expected from the previous analysis, there is a considerable difference between the velocity of colleagues and the velocity of the other groups. Friends appear to be faster than couples or families, although the difference is limited to 2-3 standard errors. We may also see that couples walk at the closest distance, followed by friends, colleagues and then families. On the other hand, families walk at the shortest abreast distance, although at a value basically equivalent to that of couples. The abreast distance of friends is significantly larger, and the one of colleagues assumes the greatest value. The “depth” *y* assumes the smallest value in couples, followed by friends and workers, and the, by a large margin, highest value in families.

#### Probability distribution functions

**Fig 6:**
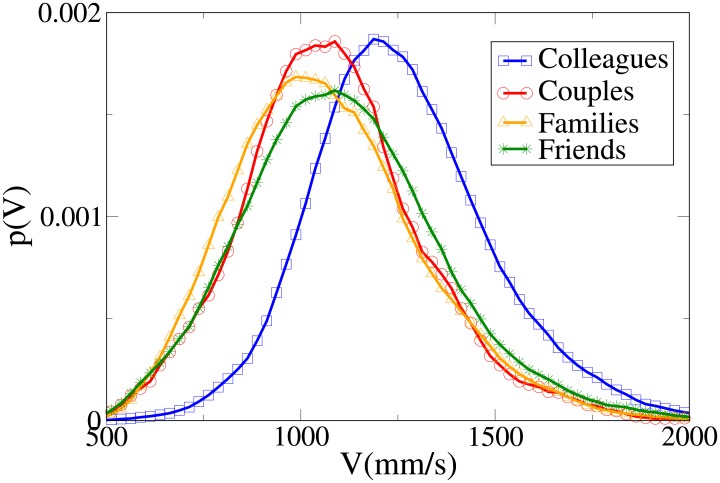
Pdf of the *V* observable according to relation. Blue squares: colleague dyads. Red circles: couples. Orange triangles: family dyads. Green stars: friend dyads.

**Fig 7:**
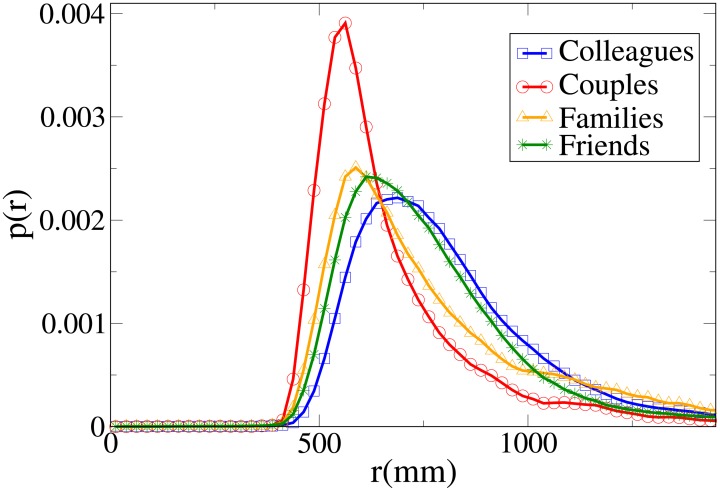
Pdf of the *r* observable according to relation. Blue squares: colleague dyads. Red circles: couples. Orange triangles: family dyads. Green stars: friend dyads.

**Fig 8:**
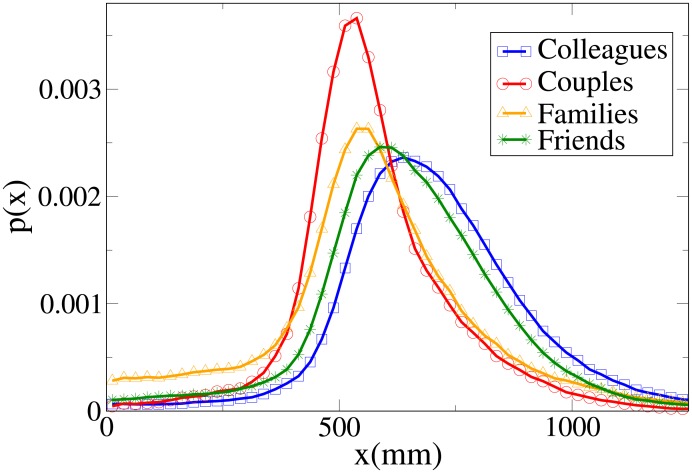
Pdf of the *x* observable according to relation. Blue squares: colleague dyads. Red circles: couples. Orange triangles: family dyads. Green stars: friend dyads.

**Fig 9:**
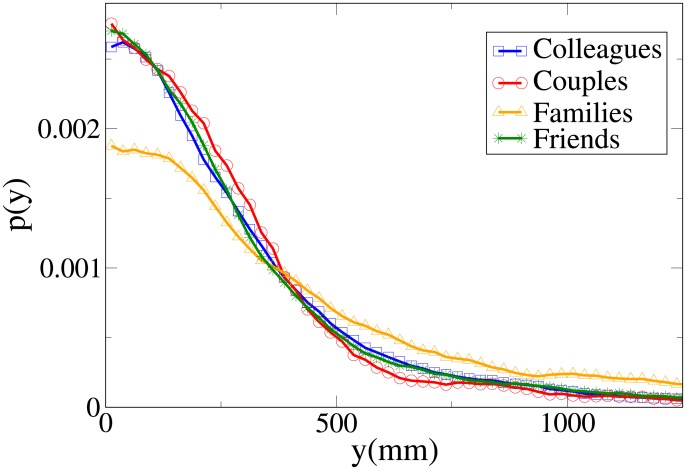
Pdf of the *y* observable according to relation. Blue squares: colleague dyads. Red circles: couples. Orange triangles: family dyads. Green stars: friend dyads.

These results may be completely understood only by analysing the probability distribution functions, which are shown in Figs 6, 7, 8 and 9 for, respectively, *V*, *r*, *x* and *y* (the statistical analysis of these distributions is reported in S2 Appendix).

The pdfs provide again an easy interpretation of the data. The *V* distributions for friends, families and couples are quite similar, while the one for colleagues is clearly different (displaced to higher values). This suggests that “relation” influences velocity in a limited way, with respect to “purpose”. The peaks of both *r* and *x* distributions assume the minimum value for couples, followed by families, friends and colleagues. The distributions for families present the following peculiar properties: the *r* distribution has a fat tail (causing the high average value), the *x* distribution assumes large values for small *x*, and the *y* distribution is more spread (on the other hand, *y* distributions are very similar in the other categories).

We may thus conclude that “relation” has an influence on distance, with couples walking at the closest distance, followed by families, friends and colleagues. At the same time, families walk in a less ordered formation (less abreast). This behaviour is probably mainly due to children (see also the analysis of the effect of age), and influences the results of the previous section (“leisure” oriented dyads walking less abreast).

#### Further analysis

In S3 Appendix we further analyse these results, to understand the effect on them of age, gender, density and height, while in S4 Appendix we verify if the major findings are confirmed by all coders. The major trends exposed above are all confirmed by this further analysis. In particular, the tendency of families to have a wider *y* distribution may be diminished but does not disappear when we keep fixed gender, age or height, showing that it is probably not only due to children.

### The effect of gender

#### Overall statistical analysis

The results related to the relation dependence for all observables concerning the 1089 dyads whose gender was coded (and that provided enough data to be analysed) are shown in Table 3.

**Table 3:** Observable dependence on gender for dyads. Lengths in millimetres, times in seconds.

Gender	$N_{g}^{k}$	*V*	*r*	*x*	*y*
Two females	252	1102 ± 12 (*σ* = 193)	790 ± 14 (*σ* = 227)	647 ± 7.8 (*σ* = 123)	321 ± 20 (*σ* = 311)
Mixed	371	1111 ± 9.5 (*σ* = 183)	824 ± 14 (*σ* = 273)	613 ± 9 (*σ* = 174)	416 ± 18 (*σ* = 350)
Two males	466	1254 ± 8.3 (*σ* = 178)	846 ± 11 (*σ* = 228)	699 ± 7.7 (*σ* = 166)	349 ± 14 (*σ* = 293)
*F*_2,1086_		84.6	4.37	30.7	7.69
*p*		< 10^−8^	0.0129	< 10^−8^	0.000484
*R*^2^		0.135	0.00798	0.0535	0.014
*δ*		0.825	0.248	0.51	0.282

#### Probability distribution functions

**Fig 10:**
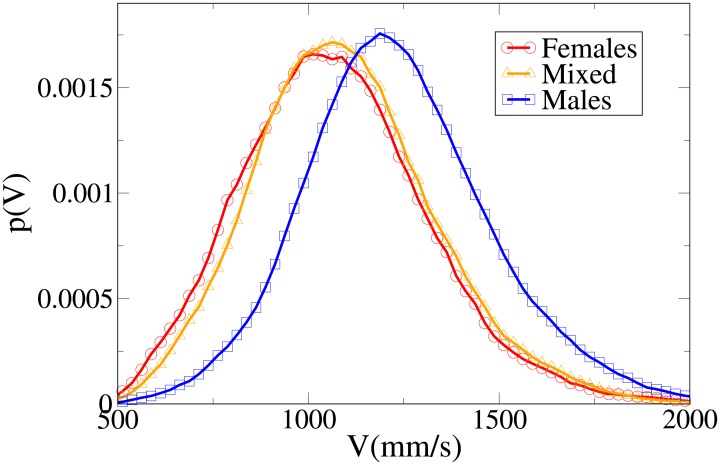
Pdf of the *V* observable in dyads according to gender. Red circles: two females. Orange triangles: mixed gender. Blue squares: two males.

**Fig 11:**
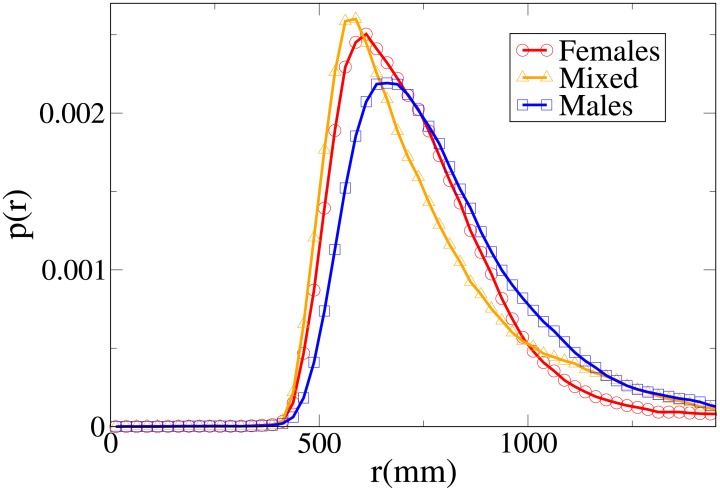
Pdf of the *r* observable in dyads according to gender. Red circles: two females. Orange triangles: mixed gender. Blue squares: two males.

**Fig 12:**
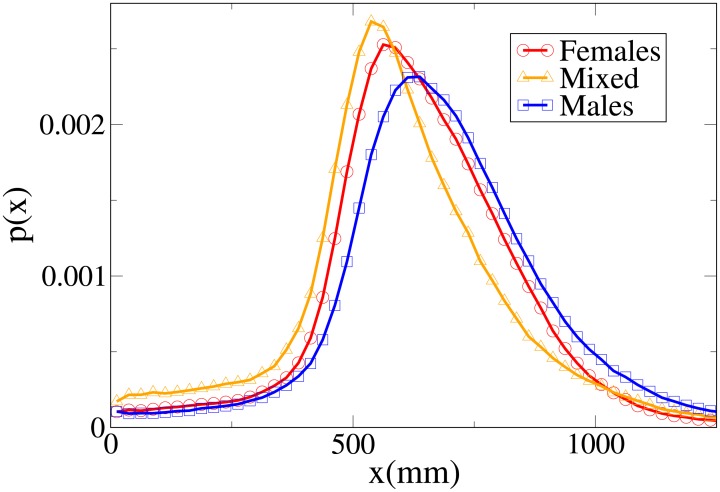
Pdf of the *x* observable in dyads according to gender. Red circles: two females. Orange triangles: mixed gender. Blue squares: two males.

**Fig 13:**
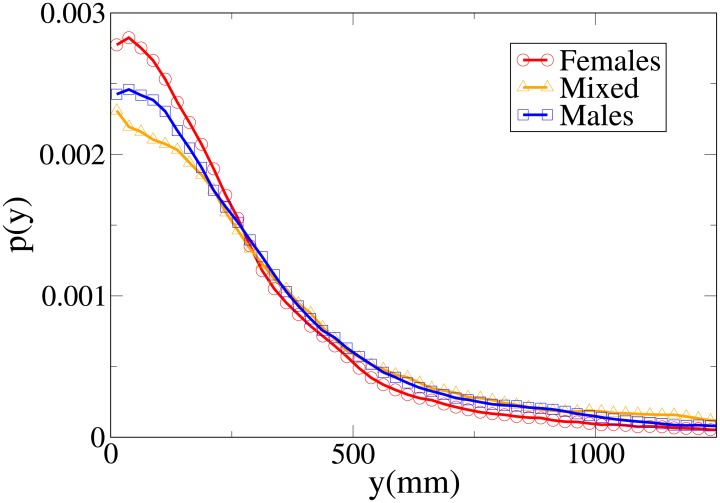
Pdf of the *y* observable in dyads according to gender. Red circles: two females. Orange triangles: mixed gender. Blue squares: two males.

Although we may easily see that the differences between the distributions are statistically significant (with stronger differences in the *V* and *x* distributions), it is again useful, in order to understand these results, to analyse the probability distribution functions, which are shown in Figs 10, 11, 12 and 13 for, respectively, the *V*, *r*, *x* and *y* observables (the statistical analysis of these distributions is reported in S2 Appendix).

The difference between the females and males distributions is very clear, with both peaks and tails in the velocity and (abreast or absolute) distance distributions displaced to higher values for males. Regarding the *y* distribution, we may see that the male distribution is more spread than the female one, and thus females have a stronger tendency to walk abreast. The mixed dyads absolute and abreast distance distribution are characterised by low values for the peaks and fat tails, in particular for the absolute value distribution. The *x* distribution presents relatively high values at low *x*, and correspondingly the *y* distribution is very spread (tendency not to walk abreast). The mixed dyads velocity distribution is interestingly very similar to the female one.

#### Further analysis

The peculiarity of the mixed distributions may be better understood by taking in consideration the other effects, in particular those related to relation, as shown in S3 Appendix. Coder reliability is analysed in S4 Appendix.

### The effect of age

To study the dependence of the *V*, *r*, *x* and *y* observables on age, we used three different approaches, namely to study how these observable change depending on *average*, *maximum* and *minimum* group age. The latter analysis appears to be the most interesting one, since it allows us to spot the presence of children, and we limit ourselves to it in the main text. Results corresponding to the dependence on average and maximum age are found in S5 Appendix.

#### Overall statistical analysis

Table 4 (and graphically S1 and S2 Figs) show the minimum age dependence of all observables (based on the analysis of 1089 dyads). Although differences between distributions are statistically significant, both velocity and distance observables are mostly constant for groups whose minimum age is in the 20-60 years range. We nevertheless find that the group depth *y* (the observable characterising thus the tendency of pedestrians not to walk abreast) assumes a very high value in groups with children, a minimum in the 20-29 years range, and then grows with age. On the other hand, abreast distance *x* is relatively low for groups with children (as we will see below, *x* grows with average height; body size could also influence the *x* distance in elderly people, due to the shorter height of elderly people in the Japanese population [54]). Velocity is mostly constant below 60 years, but drops for elderly groups.

**Table 4:** Observable dependence on minimum age for dyads. Lengths in millimetres, times in seconds.

Minimum age	$N_{g}^{k}$	*V*	*r*	*x*	*y*
0-9 years	31	1143 ± 42 (*σ* = 235)	995 ± 69 (*σ* = 383)	529 ± 34 (*σ* = 189)	701 ± 87 (*σ* = 485)
10-19 years	63	1158 ± 33 (*σ* = 259)	791 ± 33 (*σ* = 259)	624 ± 19 (*σ* = 148)	359 ± 40 (*σ* = 320)
20-29 years	364	1181 ± 9.1 (*σ* = 173)	793 ± 11 (*σ* = 218)	668 ± 8.1 (*σ* = 154)	307 ± 14 (*σ* = 264)
30-39 years	292	1204 ± 12 (*σ* = 202)	836 ± 14 (*σ* = 238)	673 ± 10 (*σ* = 176)	364 ± 18 (*σ* = 307)
40-49 years	149	1181 ± 14 (*σ* = 176)	841 ± 18 (*σ* = 224)	664 ± 13 (*σ* = 158)	384 ± 26 (*σ* = 311)
50-59 years	111	1164 ± 18 (*σ* = 193)	825 ± 21 (*σ* = 219)	649 ± 15 (*σ* = 160)	378 ± 30 (*σ* = 318)
60-69 years	67	1028 ± 21 (*σ* = 170)	881 ± 41 (*σ* = 335)	638 ± 20 (*σ* = 164)	468 ± 52 (*σ* = 422)
≥ 70 years	12	886 ± 29 (*σ* = 99.8)	786 ± 79 (*σ* = 275)	588 ± 19 (*σ* = 66.6)	385 ± 100 (*σ* = 363)
*F*_7,1081_		10.7	3.96	4.23	8.02
*p*		< 10^−8^	0.000282	0.000128	< 10^−8^
*R*^2^		0.065	0.025	0.0267	0.0494
*δ*		1.6	0.583	0.808	1.37

#### Probability distribution functions

**Fig 14:**
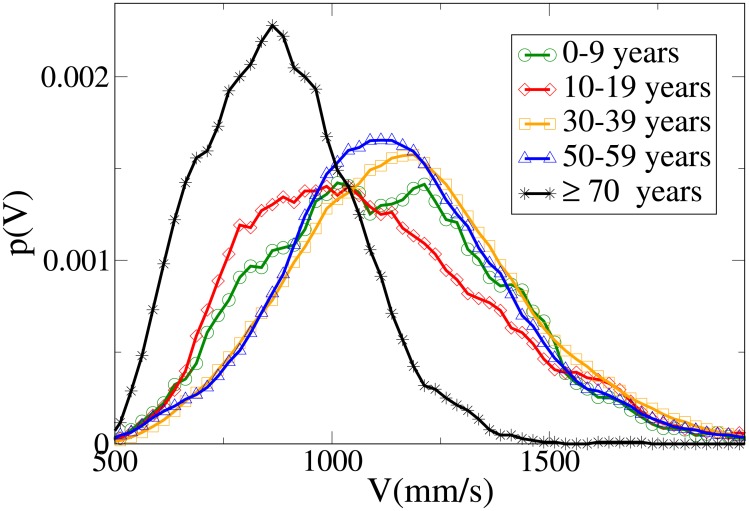
Probability distribution function for *V* according to minimum age. Green circles: 0-9 years range. Red diamonds: 10-19 years range. Orange squares: 30-39 years range. Blue triangles: 50-59 years range. Black stars: over 70 years range.

**Fig 15:**
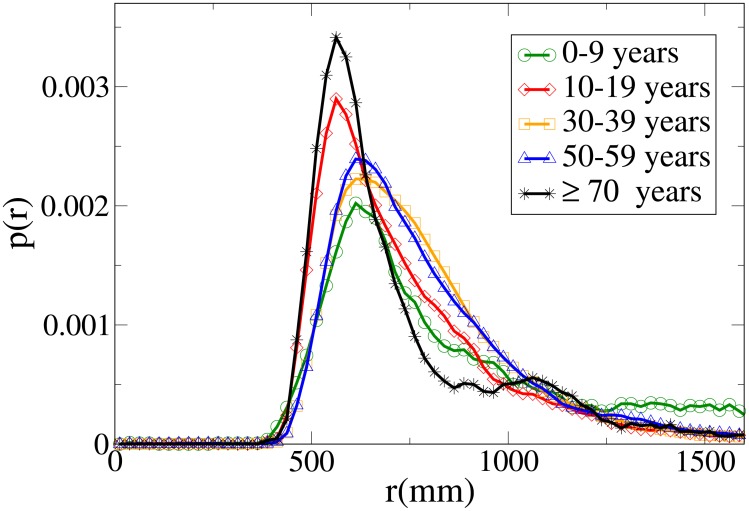
Probability distribution function for *r* according to minimum age. Green circles: 0-9 years range. Red diamonds: 10-19 years range. Orange squares: 30-39 years range. Blue triangles: 50-59 years range. Black stars: over 70 years range.

**Fig 16:**
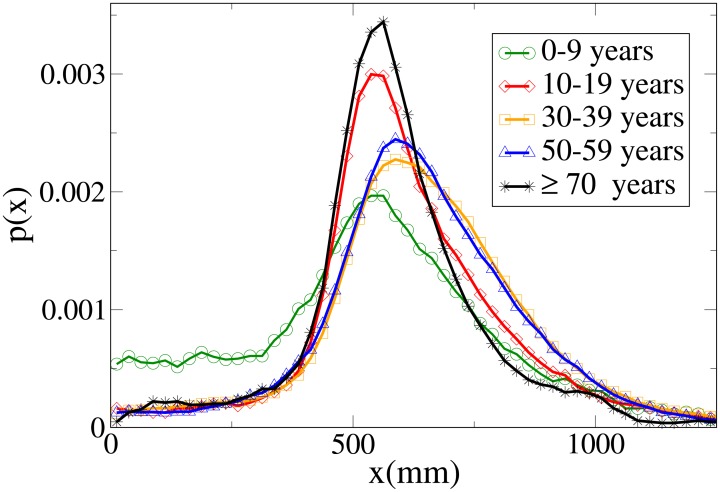
Probability distribution function for *x* according to minimum age. Green circles: 0-9 years range. Red diamonds: 10-19 years range. Orange squares: 30-39 years range. Blue triangles: 50-59 years range. Black stars: over 70 years range.

**Fig 17:**
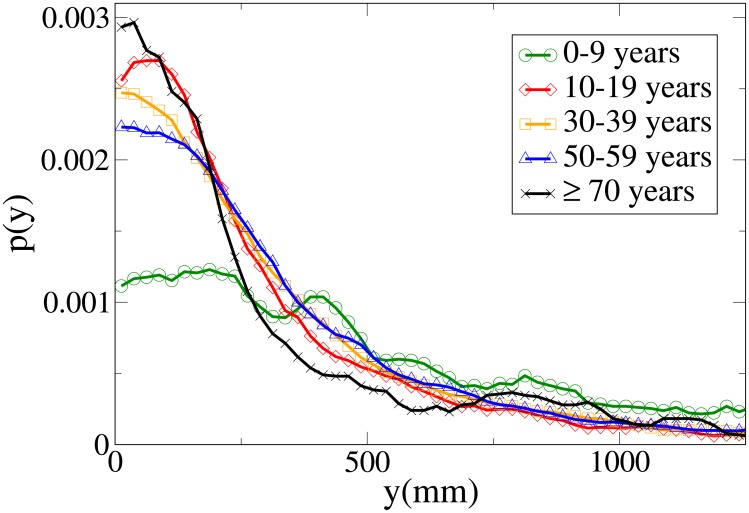
Probability distribution function for *y* according to minimum age. Green circles: 0-9 years range. Red diamonds: 10-19 years range. Orange squares: 30-39 years range. Blue triangles: 50-59 years range. Black stars: over 70 years range.

The probability functions for different observables in different age ranges are shown in Figs 14, 15, 16 and 17 respectively for observables *V*, *r*, *x* and *y*, and their statistical analysis is presented in S2 Appendix. We may easily see from the large tail of the *r* distribution, the high values for the *x* distribution and the spread of the *y* distribution, that the presence of a child causes the group not to walk very abreast. The abreast distance peak is higher in “working age people”with respect to young and elderly dyads. Elderly people have a very narrow peak in the *y* distribution, but also a fat tail. Velocity in the 0-19 age range assumes lower peaks than in the 20-59 range, but has a large spread, while in elderly people it assumes clearly lower values. As stated in the Materials and methods section, the tracking of short people (and thus of children) is more difficult, and as a consequence the tracked position could be affected by higher sensor noise, although our time filter (see again the Materials and methods section) should remove this problem. We thus examined a portion of the videos corresponding to group with children, and noticed that children have indeed an erratic behaviour that leads them to sudden accelerations and non-abreast formations. We thus believe that the large spread of observables for dyads with children is due to actual pedestrian behaviour.

#### Further analysis

In S3 Appendix we analyse possible effects due to density, relation, gender and age. Interesting results reported in the appendix suggest a tendency of families *not* to walk abreast even when formed only by adults (this could be related to a visual bias of coders, that code mixed dyads as families when not walking abreast, and couples when walking abreast), and differences in groups with children based on gender (probably affected by the gender of the parent). Coder reliability is analysed in S4 Appendix.

A further interesting result is that, as shown in S3 Fig, dyads with children *walk faster at higher density*, in contrast with the usual pedestrian behaviour.

### The effect of height

Height is the only pedestrian feature that is not the result of coding, since it is automatically tracked by our system [43]. We again considered (see S6 Appendix) average, minimum and maximum height. The three indicators give similar results, and in the following we use minimum height to better identify the presence of children.

#### Overall statistical analysis

The dependence of all observables on minimum height (based on 1089 dyads) is shown in Table 5. We have significant statistical difference for all observables, but the interpretation of the results is not straightforward, due to the peculiar behaviour of dyads including short people (most probably children). From Table 5, and graphically from S4 Fig, we see that velocity grows (as expected, see for example [36] and [5]), with height, but dyads with a very short individual represent an exception (children move fast despite the short height). Again from the data in Table 5 or from S5 Fig we may see that distance is mostly independent of height above 150 cm, but assumes a very high value for dyads including short pedestrians. The height dependence of *y*, shown graphically also in S6 Fig, results to be a decreasing function, although a comparison with a linear fit shows that dyads including people under 140 cm walk with a particularly spread (non-abreast) *y* distribution, while above 150 cm the group depth is almost constant. The *x* observable, on the other hand, appears to grow mostly in a linear way (see also S7 Fig). This could lead us to think that abreast distance depends only on body size. Nevertheless, while there is probably a strong dependence of abreast distance on height, this linear dependence is also due to the balance between the male and female behaviour, which, when examined separately, exhibit non-linear dependence on height, as shown in S8 Fig. Furthermore, as we will see in the “Probability distribution functions” subsection below, the growth in *x* with height is a combination of a increase of peak position and decrease of people walking in non-abreast formation.

**Table 5:** Observable dependence on minimum height for dyads. Lengths in millimetres, times in seconds.

Minimum height	$N_{g}^{k}$	*V*	*r*	*x*	*y*
< 140 cm	39	1130 ± 34 (*σ* = 211)	1004 ± 65 (*σ* = 404)	573 ± 34 (*σ* = 210)	672 ± 80 (*σ* = 501)
140-150 cm	39	1106 ± 50 (*σ* = 311)	875 ± 46 (*σ* = 289)	619 ± 25 (*σ* = 156)	469 ± 64 (*σ* = 403)
150-160 cm	234	1104 ± 13 (*σ* = 197)	797 ± 16 (*σ* = 246)	631 ± 8.9 (*σ* = 136)	360 ± 21 (*σ* = 328)
160-170 cm	498	1169 ± 8.1 (*σ* = 182)	821 ± 11 (*σ* = 243)	657 ± 7.7 (*σ* = 172)	362 ± 14 (*σ* = 311)
170-180 cm	262	1242 ± 11 (*σ* = 173)	827 ± 12 (*σ* = 197)	699 ± 9.6 (*σ* = 155)	321 ± 16 (*σ* = 251)
> 180 cm	17	1232 ± 51 (*σ* = 211)	793 ± 48 (*σ* = 198)	689 ± 33 (*σ* = 135)	270 ± 53 (*σ* = 217)
*F*_5,1083_		14.5	5.25	7.45	9.69
*p*		< 10^−8^	9.03⋅10^−5^	6.9⋅10^−7^	< 10^−8^
*R*^2^		0.0626	0.0237	0.0333	0.0428
*δ*		0.744	0.591	0.773	0.922

#### Probability distribution functions

**Fig 18:**
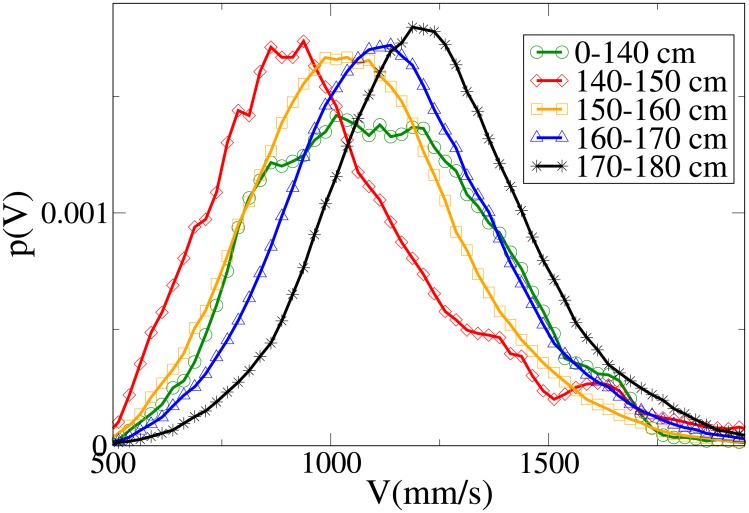
Probability distribution function for *V* according to minimum height. Green circles: 0-140 cm range. Red diamonds: 140-150 cm range. Orange squares: 150-160 cm range. Blue triangles: 160-170 cm range. Black stars: 170-180 cm range.

**Fig 19:**
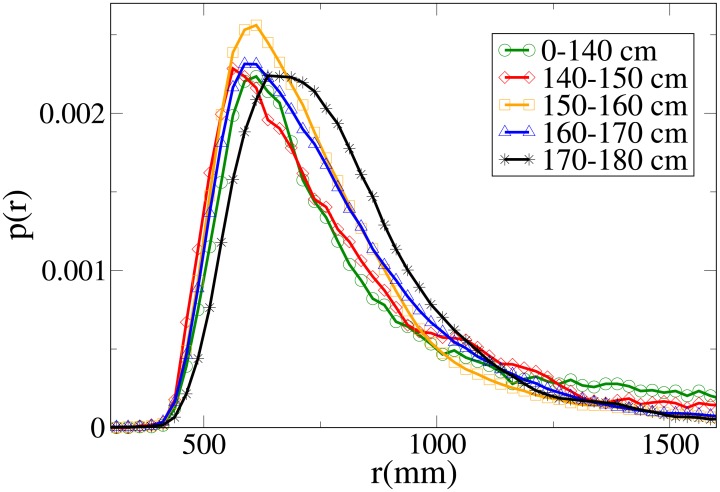
Probability distribution function for *r* according to minimum height. Green circles: 0-140 cm range. Red diamonds: 140-150 cm range. Orange squares: 150-160 cm range. Blue triangles: 160-170 cm range. Black stars: 170-180 cm range.

**Fig 20:**
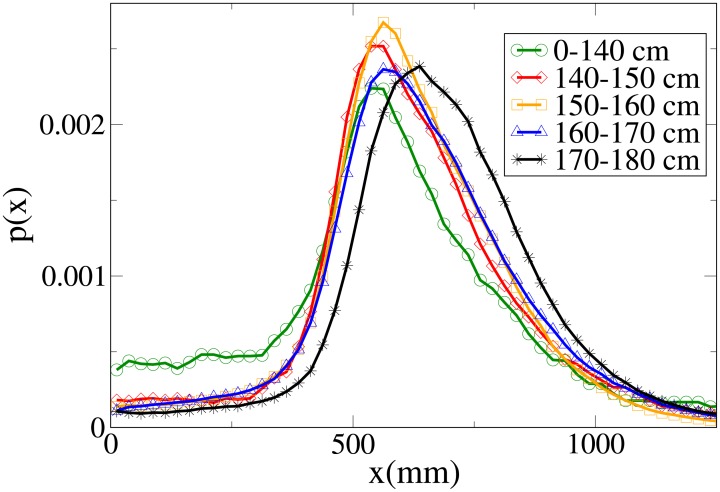
Probability distribution function for *x* according to minimum height. Green circles: 0-140 cm range. Red diamonds: 140-150 cm range. Orange squares: 150-160 cm range. Blue triangles: 160-170 cm range. Black stars: 170-180 cm range.

**Fig 21:**
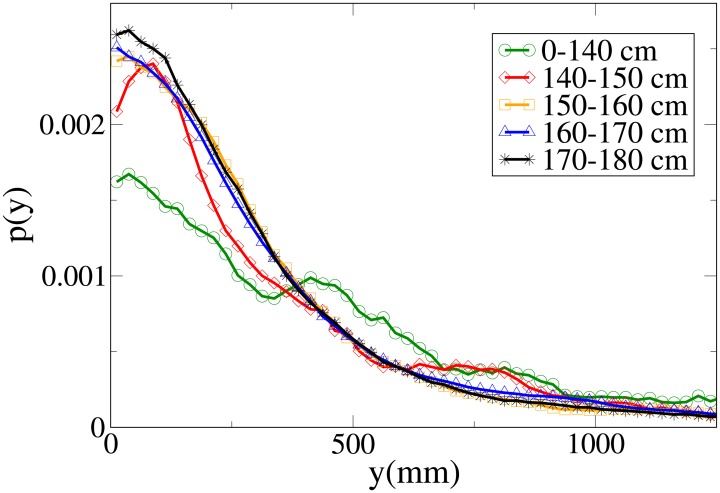
Probability distribution function for *y* according to minimum height. Green circles: 0-140 cm range. Red diamonds: 140-150 cm range. Orange squares: 150-160 cm range. Blue triangles: 160-170 cm range. Black stars: 170-180 cm range.

The probability functions for different observables in different minimum height ranges are shown in Figs 18, 19, 20 and 21, respectively for the *V*, *r*, *x* and *y* observables, and their statistical analysis is shown in S2 Appendix.

We see, as stated in the previous subsection, that the abreast distance distributions are displaced to the right with growing height, with a corresponding decrease in the values assumed around zero (particularly high in the 0-140 cm distribution, probably due to children behaviour). Similarly, the *y* distribution becomes narrower with growing height, and presents a very different behaviour in the shortest height slot. The absolute distance distributions are displaced to the right with growing height, but the very fat tail for the 0-140 cm distribution causes the average value to have a more complex dependence on height. The *V* distribution shows a clear displacement to the right with growing height, both in peaks and tails, although the 0-140 cm distribution has again a peculiar behaviour due to its very pronounced width.

### Further analysis

In S3 Appendix we analyse the validity of these results on height dependence when we consider other effects such as age, relation, gender and density, and verify that, although sometimes diminished, height related results are present also when analysing groups with fixed age, relation and gender.

## Discussion and conclusion

### Summary of our findings

In this work we analysed how pedestrian dyad behaviour depends on the group’s “intrinsic properties”, namely the characteristics of its members and the relation between them. We observed that female dyads are slower and walk closer than males, that workers walk faster, at a larger distance and more abreast than leisure oriented people, and that inter-group relation has a strong effect on group structure, with couples walking very close and abreast, colleagues walking at a larger distance, and friends walking more abreast than family members. Pedestrian height influences velocity and abreast distance, observables that grow with the average or minimum group height. We also found that elderly people walk slowly, while active age adults walk at the maximum velocity. Dyads with children have a strong tendency to walk in a non-abreast formation, with a large distance but a shorter abreast distance.

Our results are also qualitatively summarised, for each observable and feature, in Table 6.

**Table 6:** Qualitative summary of our results.

	Speed *V*	Distance *r*	Extension *x*	Depth *y*
Purpose	faster in workers	larger in workers	larger in workers	smaller in workers
Relation	fastest in colleagues	largest in families, followed by colleagues	smallest in families and couples	largest in families, smallest in couples
Gender	fastest in males	largest in males	largest in males	largest in mixed groups
Age	fastest in active adults	largest in groups with children	largest in active adults	largest in groups with children
Height	growing with height	largest in short people (children)	growing with height	largest in short people (children)

In the supporting material appendices, we analysed how these features affect each other, and we verified that the effects of the different features are present, even though sometimes diminished, when the other features are kept fixed (e.g., when we compare colleagues of different gender, and the like). This cross-analysis helps in better clarifying some effects that are not completely clear when a single feature is considered. For example, we have found that the tendency of families *not to walk very abreast* (large *y*) is indeed stronger when children are present, but appears to be an authentic family effect (i.e. present also in adult families). The peculiar behaviour of mixed gender groups, on the other hand, is better clarified by analysing its dependence on relation, and finding that there is not a “typical mixed group” (couples behave differently from mixed colleague groups which are again different from mixed families and mixed friend groups).

Two particularly interesting and surprising results in the cross-analysis were the findings that the velocity of dyads with children appears to increase with density (at least in the low-medium density range), and that children behaviour appears to be influenced by the gender of the parent. The first result seems to suggest that children behaviour becomes particularly erratic in more crowded settings (which in the studied environment correspond mainly to non-working days), while the second one seems to suggest that, in this cultural setting, children walk faster but also more abreast, and thus in a more ordered way, when the father is present. These two results are based on an extremely reduced amount of data, but suggest an interesting direction for future studies.

In this work we focused on “group features” more than “individual features”, i.e. we did not explicitly address questions such as the age or height difference, and similar. We may nevertheless infer from our results some information about how group members with different height, age and gender “compromise” on group dynamics. We may see, for example, in S6 Appendix that average age gives, for the *V* and *x* observables that are growing functions of height, a result in between those obtained for minimum and maximum height, i.e. the pedestrians in the group appear to choose a velocity which is an average between the individual ones. On the other hand, gender and age appear to have a deeper and less trivial impact on social interactions. In mixed groups, males appear to adapt to female velocity when we average over relations, but when we analyse for secondary effects in S3 Appendix, we see that this is true for couples and families, but it does not apply to friends or colleagues. Similarly, while couples walk closer than male or female same sex dyads, mixed colleague groups walk farther than same sex dyads of both genders. In a similar way, due also to the peculiar behaviour of children, it is impossible to find a simple “compromise” rule for age related behaviour. More information could be inferred by an analysis taking in explicit account age differences, that we reserve for a future work.

The exact figures found in this work may depend strongly on the environment in which they have been recorded, and vary not only with density, but also with other macroscopic crowd dynamics features (uni-directional flow, bi-directional flow, multi-directional flow, presence or not of standing pedestrians, etc.) as well as architectural features of the environment (open space or large corridor or narrow corridor, etc.). For this reason, attempts to verify our findings in different environments should be directed not at specific quantitative figures (e.g. male dyads walk at 1.25 m/s and females at 1.1 meters per second) but at qualitative patterns (e.g. males walk faster than females in a statistically significant way, with a difference in velocity comparable to the standard deviation in distributions).

### Future work

A possible extension of this work regards the analysis of three people group behaviour. Furthermore, as stated above, in this work we limited ourselves to group properties and not individual properties (e.g., we verified if a group was mixed, but we did not study the specific position of the male or female). After a revision of the coding procedure, we could analyse if, according to gender, age or height differences, roles such as “leader” or “follower” emerge. Finally, a mathematical modelling following [3] and [33] could be performed. It would also be very interesting to compare our findings with different cultural settings, since it may be expected that social group behaviour is strongly dependent on culture, so that at least some of the patterns could change when similar data collection experiments are performed outside of (western) Japan.

### Possible technological impact

Besides the obvious applications to pedestrian simulations, with possible influence in building and events planning, disaster prevention, and even in entertainment industries such as movies and video games, we are particularly interested in applications in the field of human robot interaction [55] and in the development of slow vehicles with automatic navigation capabilities deployed in pedestrian facilities, such as delivery vehicles or automatic wheelchairs and carts [56]. Such vehicles will arguably become more common in the future, and in order to navigate safely inside human crowds, and to move together with other humans “as in a group”, they will need an understanding of pedestrian and group behaviour.

More specifically, a “companion” robot or an automatic wheelchair will need to be able to:

1. recognise pedestrian groups, using an automatic recognition algorithm [57, 58],
2. predict their behaviour, both in order to be able to safely avoid them and to perform a socially acceptable behaviour [59],
3. move together with other humans, and behave as a member of a group [60].

For all these applications it is extremely important to understand deeply how pedestrians actually behave and we plan to use the findings of this work to improve our previous algorithms and systems as part of the development of a platform for autonomous personal mobility systems.

## Supporting information

S1 Data Set Group trajectories and coding data set. (ZIP)

S1 Fig *V* dependence on minimum age. Dashed lines provide standard error confidence intervals. The point at 75 years corresponds to the “70 years or more” slot.
(EPS)

S2 Fig *r*, *x* and *y* dependence on minimum age. Black circles: *r*; red squares: *x*; blue triangles: *y*. Dashed lines provide standard error confidence intervals. The point at 75 years corresponds to the “70 years or more” slot.
(EPS)

S3 Fig Dependence of *V* on minimum age at different densities. Blue squares: 0 ≤ *ρ* < 0.05 ped/m^2^ range; red circles: 0.15 ≤ *ρ* < 0.2 ped/m^2^ range. The point at 75 years corresponds to the “70 years or more” slot. Dashed lines show standard error confidence intervals.
(EPS)

S4 Fig *V* dependence on minimum height. Data points shown by red circles. Continuous black line: linear best fit with *V* = *α* + *βh*, *α* = 715 mm/s, *β* = 2.81 s^−1^. Dashed lines provide standard error confidence intervals, the point at 135 cm corresponds to the “less than 140 cm” slot, the one at 185 cm to the “more than 180 cm” slot.
(EPS)

S5 Fig *r* dependence on minimum height. Data points shown by red circles. Continuous black line: linear best fit with *r* = *α* + *βh*, *α* = 1390 mm, *β* = -3.35. Dashed lines provide standard error confidence intervals, the point at 135 cm corresponds to the “less than 140 cm” slot, the one at 185 cm to the “more than 180 cm” slot.
(EPS)

S6 Fig *y* dependence on minimum height. Data points shown by red circles. Continuous black line: linear best fit with *y* = *α* + *βh*, *α* = 1530 mm, *β* = -7.01. Dashed lines provide standard error confidence intervals, the point at 135 cm corresponds to the “less than 140 cm” slot, the one at 185 cm to the “more than 180 cm” slot.
(EPS)

S7 Fig *x* dependence on minimum height. Data points shown by red circles. Continuous black line: linear best fit with *x* = *α* + *βh*, *α* = 258 mm, *β* = 2.42. Dashed lines provide standard error confidence intervals, the point at 135 cm corresponds to the “less than 140 cm” slot, the one at 185 cm to the “more than 180 cm” slot.
(EPS)

S8 Fig *x* dependence on minimum height for different genders. Red circles: two females; blue squares: two males (continuous lines: linear fits *x* = *α* + *βh*, *α*_female_ = 154 mm, *β*_female_ = 2.98; *α*_male_ = 211 mm, *β*_male_ = 2.79). The points at 135 and 185 cm represent the “less than 140” and “more than 180” cm slots). Dashed lines show confidence intervals.
(EPS)

S1 Appendix Statistical analysis of observables. (PDF)

S2 Appendix Statistical analysis of overall probability distributions. (PDF)

S3 Appendix Accounting for other effects. (PDF)

S4 Appendix Coder reliability. (PDF)

S5 Appendix Dependence on average and maximum age. (PDF)

S6 Appendix Comparison between minimum, average and maximum height. (PDF)

## References

[1] Challenger W, Clegg WC and Robinson AM, *Understanding crowd behaviours: guidance and lessons identified*, UK Cabinet Office, 11–13, 2009.

[2] Reicher SD, *The psychology of crowd dynamics*, Vol. 44 No. 0 Blackwell handbook of social psychology: Group processes, 2001.

[3] Zanlungo F, Ikeda T, and Kanda T, Potential for the dynamics of pedestrians in a socially interacting group, Physical Review E, 2014, 89, 1, 021811 doi: 10.1103/PhysRevE.89.012811 10.1103/PhysRevE.89.012811 24580285

[4] Zanlungo F, Brščić D, and Kanda T, Spatial-size scaling of pedestrian groups under growing density conditions, Physical Review E, 2015, 91 (6), 062810 doi: 10.1103/PhysRevE.91.062810 10.1103/PhysRevE.91.062810 26172757

[5] Brščić D, Zanlungo F, and Kanda T, Density and velocity patterns during one year of pedestrian tracking, Transportation Research Procedia, 2014, 2, 77–86. doi: 10.1016/j.trpro.2014.09.011

[6] Schultz M, Rößger L, Hartmut F, and Schlag B, Group dynamic behavior and psychometric profiles as substantial driver for pedestrian dynamics, in *Pedestrian and Evacuation Dynamics 2012*, Weidmann U, Kirsh U, and Schreckenberg M editors, Vol II, pp. 1097–1111; Springer; 2014.

[7] Moussaïd M, Perozo N, Garnier S, Helbing D, and Theraulaz G, The walking behaviour of pedestrian social groups and its impact on crowd dynamics, PLoS One, 2010, 5, 4, e10047 doi: 10.1371/journal.pone.0010047 20383280 10.1371/journal.pone.0010047 PMC2850937

[8] Mawson AR, *Mass panic and social attachment: the dynamics of human behavior*, Ashgate Publishing, Ltd, 2012.

[9] He L, Pan J, Wang W and Manocha, D, *Proxemic group behaviors using reciprocal multi-agent navigation*, 2016 IEEE International Conference on Robotics and Automation (ICRA), 292–297.

[10] Kumagai K, Ehiro I, and Ono K, *Numerical simulation model of group walking for tsunami evacuees*, Proceedings of the 2016 Pedestrian and Evacuation Dynamics Conference.

[11] Kachroo P, Al-Nasur SJ, Wadoo SA, and Shende A, *Pedestrian dynamics: feedback control of crowd evacuation*, Springer Science & Business Media, 2008.

[12] Seitz MJ, Templeton A, Drury J, Köster G, and Philippides A, Parsimony versus reductionism: how can crowd psychology be introduced into computer simulation?, Review of General Psychology, 21(1), 95, 2017 doi: 10.1037/gpr0000092

[13] Kim, S, Guy, SJ, Manocha D, and Lin, MC *Interactive simulation of dynamic crowd behaviors using general adaptation syndrome theory*, Proceedings of the ACM SIGGRAPH Symposium on Interactive 3D Graphics and Games, ACM, 2012.

[14] Costa M, Interpersonal distances in group walking, Journal of Nonverbal Behavior, 2010, 34, 1, 15–26. doi: 10.1007/s10919-009-0077-y

[15] Zanlungo F, and Kanda T, *Do walking pedestrians stabily interact inside a large group? Analysis of group and sub-group spatial structure*, COGSCI13 (2013 Cognitive Science Society Conference).

[16] Gorrini A, Vizzari G, and Bandini S, *Granulometric distribution and crowds of groups: focusing on dyads*, 11th Conference International Traffic and Granular Flow, Delft (NL), (2015)

[17] Bandini S, Crociani L, Gorrini A, and Vizzari G, *An agent-based model of pedestrian dynamics considering groups: A real world case study*, IEEE 17th International Conference on Intelligent Transportation Systems (ITSC), 2014, 572–577.

[18] Köster G, Seitz M, Treml F, Hartmann D, and Klein W, On modeling the influence of group formation in a crowd, Contemporary Social Science, 2011, 6, 3, 397–414. doi: 10.1080/21582041.2011.619867

[19] Köster G, Treml F, Seitz M, and Klein W, Validation of crowd models including social groups, In *Pedestrian and Evacuation Dynamics 2012*, Weidmann U, Kirsh U, and Schreckenberg M editors, Vol II, pp. 1051–1063; Springer; 2014.

[20] Wei X, Lv X, Song W, and Li X, Survey study and experimental investigation on the local behavior of pedestrian groups, Complexity, Volume 20, Issue 6 7/8 2015, pp. 87–97. doi: 10.1002/cplx.21633

[21] Karamouzas I, and Overmars M, *Simulating the local behaviour of small pedestrian groups*, Proceedings of the 2010 17th ACM Symposium on Virtual Reality Software and Technology, 183–190.

[22] Zhang Y, Pettré J, Qin X, Donikian S, and Peng Q, *A local behavior model for small pedestrian groups*, 12th International Conference on Computer-Aided Design and Computer Graphics (CAD/Graphics), 2011, 275–281.

[23] Bode N, Holl S, Mehner W, and Seyfried A, Disentangling the impact of social groups on response times and movement dynamics in evacuations, PLoS one http://dx.doi.org/10.1371/journal.pone.0121227 10.1371/journal.pone.0121227 PMC4364745 25785603

[24] Gorrini A, Crociani L, Feliciani C, Zhao P, Nishinari K, and Bandini S, *Social groups and pedestrian crowds: experiment on dyads in a counter flow scenario*, Proceedings of the 2016 Pedestrian and Evacuation Dynamics Conference.

[25] Zhao P, Sun L, Cui L, Luo W, and Ding Y, *The walking behaviours of pedestrian social group in the corridor of subway station*, Proceedings of the 2016 Pedestrian and Evacuation Dynamics Conference.

[26] von Krüchten C, Müller F, Svachiy A, Wohak O, and Schadschneider A, Empirical study of the influence of social groups in evacuation scenarios, Traffic and Granular Flow’15 (Springer, 2016)

[27] Wang W, Lo S, Liu S, and Ma J, *A simulation of pedestrian side-by-side walking behaviour and its impact on crowd dynamics*, Proceedings of the 2016 Pedestrian and Evacuation Dynamics Conference.

[28] Huang J, Zou X, Qu X, Ma J, and Xu R, *A structure analysis method for complex social pedestrian groups with symbol expression and relationship matrix*, Proceedings of the 2016 Pedestrian and Evacuation Dynamics Conference.

[29] Manfredi M, Vezzani R, Calderara S and Cucchiara R, Detection of static groups and crowds gathered in open spaces by texture classification, Pattern Recognition Letters, 44, 39–48 (2014). doi: 10.1016/j.patrec.2013.11.001

[30] von Krüchten C, and Schadschneider A, Empirical study on social groups in pedestrian evacuation dynamics, Physica A: Statistical Mechanics and its Applications, 475, 129–141, (2017) doi: 10.1016/j.physa.2017.02.004

[31] Faria JJ, Dyer JR, Tosh CR and Krause J, Leadership and social information use in human crowds, Animal Behaviour, 79(4), 895–901 (2010) doi: 10.1016/j.anbehav.2009.12.039

[32] Templeton A, Drury J and Philippides A, From mindless masses to small groups: conceptualizing collective behavior in crowd modeling, Review of General Psychology, 19(3), 215 (2015). doi: 10.1037/gpr0000032 26388685 10.1037/gpr0000032 PMC4568938

[33] Zanlungo F, and Kanda T, A mesoscopic model for the effect of density on pedestrian group dynamics, EPL (Europhysics Letters), 2015; 111, 38007 doi: 10.1209/0295-5075/111/38007

[34] Turchetti G, Zanlungo F, Giorgini G, Dynamics and thermodynamics of a gas of automata, EPL (Europhysics Letters) 78 (5), 58003 doi: 10.1209/0295-5075/78/58003

[35] Zanlungo F, Brščić D, and Kanda T, Pedestrian group behaviour analysis under different density conditions, Transportation Research Procedia, 2014, 2, 149–158. doi: 10.1016/j.trpro.2014.09.020

[36] Bohannon R, Comfortable and maximum walking speed of adults aged 20–79 years: reference values and determinants, Age and ageing, 1997, 26, 15–19. doi: 10.1093/ageing/26.1.15 9143432 10.1093/ageing/26.1.15

[37] Bode N, *The effect of social groups and gender on pedestrian behaviour immediately in front of bottlenecks*, Proceedings of the 2016 Pedestrian and Evacuation Dynamics Conference.

[38] Henderson L, and Lyons D, Sexual differences in human crowd motion, Nature 240, 08 12 1972, 353–355. doi: 10.1038/240353a0 4570498 10.1038/240353a0

[39] von Sivers I, Künzner F, and Köster G, *Pedestrian evacuation simulation with separated families*, Proceedings of the 2016 Pedestrian and Evacuation Dynamics Conference.

[40] Feng Y, and Li D, *An empirical study and conceptual model on heterogenity of pedestrian social groups for friend -group and family-group*, Proceedings of the 2016 Pedestrian and Evacuation Dynamics Conference.

[41] Müller F, and Schadschneider A, Evacuation dynamics of asymmetrically coupled pedestrian pairs, Traffic and Granular Flow’15 (Springer, 2016).

[42] Zanlungo F, Yücel Z, and Kanda T, *The effect of social roles on group behaviour*, Proceedings of the 2016 Pedestrian and Evacuation Dynamics Conference.

[43] Brščić D, Kanda T, Ikeda T, and Miyashita T, Person tracking in large public spaces using 3-D range sensors, IEEE Transactions on Human Machine Systems, 2013, vol 43, no 6, pp 522–534. doi: 10.1109/THMS.2013.2283945

[44] Bandini S, Gorrini A and Nishinari K, *Crossing disciplinary borders through studying walkability*, In International Conference on Cross-Cultural Design (pp. 491–503), Springer International Publishing (2016)

[45] Levine RV and Norenzayan A, The pace of life in 31 countries. *Journal of cross-cultural psychology*, 30(2), 178–205, (1999). doi: 10.1177/0022022199030002003

[46] http://www.irc.atr.jp/sets/groups/.

[47] Seer S, Brändle N, and Ratti C, Kinects and human kinetics: a new approach for studying crowd behavior, Transportation Research Part C: Emerging Technologies, 2014, 48(0):212–228. doi: 10.1016/j.trc.2014.08.012

[48] Corbetta A, Bruno L, Muntean A, and Toschi F, High statistics measurements of pedestrian dynamics, Transportation Research Procedia, 2014, 2, 96–104. doi: 10.1016/j.trpro.2014.09.013

[49] Knapp M, Nonverbal communication in human interaction, Cengage Learning; 2012.

[50] Kleinke C, Gaze and eye contact: a research review., Psychological bulletin, 1986, 100, 1, 78 doi: 10.1037/0033-2909.100.1.78 3526377

[51] Argyle M, and Dean J, Eye-contact, distance and affiliation, Sociometry, 1965, 289–304. doi: 10.2307/2786027 14341239

[52] Zhang J, Klingsch W, Schadschneider A, and Seyfried A, Transitions in pedestrian fundamental diagrams of straight corridors and T-junctions, Journal of Statistical Mechanics: Theory and Experiment, 2011, 06, P06004.

[53] Corbetta A, Jasper M, Lee C, and Toschi F, *Continuous measurements of real-life bidirectional pedestrian flows on a wide walkway*, Proceedings of the 2016 Pedestrian and Evacuation Dynamics Conference.

[54] http://www.mext.go.jp/b_menu/toukei/001/022/2004/002.pdf (in Japanese).

[55] Kanda T, Shiomi M, Miyashita Z, Ishiguro H, and Hagita N, *An affective guide robot in a shopping mall*, 2009 ACM/IEEE International Conference on Human-Robot Interaction (HRI), 173–180.

[56] Morales Y, Kallakuri N, Shinozawa K, Miyashita T and Hagita N., *Human-comfortable navigation for an autonomous robotic wheelchair*, 2013 IEEE/RSJ International Conference on Intelligent Robots and Systems (IROS).

[57] Yücel Z, Zanlungo F, Ikeda T, Miyashita T, and Hagita N, Deciphering the crowd: modeling and identification of pedestrian group motion, Sensors, 2013, vol. 13, pp. 875–897. doi: 10.3390/s130100875 23344382 10.3390/s130100875 PMC3574710

[58] Brščić D, Zanlungo F, and Kanda T, *Modelling of pedestrian groups and application to group recognition*, 2017 40th International Convention on Information Information and Communication Technology, Electronics and Microelectronics (MIPRO), Opatija, 2017, pp. 564–569.

[59] Shiomi M, Zanlungo F, Hayashi K, and Kanda T, Towards a socially acceptable collision avoidance for a mobile robot navigating among pedestrians using a pedestrian model, International Journal of Social Robotics, 2014, 6, 3, 443–455. doi: 10.1007/s12369-014-0238-y

[60] Murakami R, Morales Saiki LY, Satake S, and Kanda T, *Destination unknown: walking side-by-side without knowing the goal*, Proceedings of the 2014 ACM/IEEE international conference on Human-robot interaction, 471–478.

