# Supplementary material for: Intrinsic group behaviour: Dependence of pedestrian dyad dynamics on principal social and personal features
Source: PLoS One. 2017 Nov 2;12(11):e0187253. doi: 10.1371/journal.pone.0187253 (PMC5667819; doi:10.1371/journal.pone.0187253)
Supplement: S2 Appendix — (PDF) [file pone.0187253.s011.pdf]

Statistical analysis of overall probability distributions

Purpose

Table 1 provides a statistical analysis of the overall probability distributions for the purpose categories.

Table 1: Statistical analysis of the overall probability distributions for the purpose categories. Lengths in millimetres, times in seconds.

Purpose	N^k	V	r	x	y
Leisure	38501	1096 ± 1.3 (σ=251)	799 ± 1.6 (σ=309)	630 ± 1.2 (σ=236)	360 ± 2 (σ=388)
Work	18936	1257 ± 1.7 (σ=235)	834 ± 2.1 (σ=287)	714 ± 1.6 (σ=227)	315 ± 2.5 (σ=341)
$F_{1,57435}$		5400	169	1640	184
p		$< 10^{-8}$	$< 10^{-8}$	$< 10^{-8}$	$< 10^{-8}$
R^2		0.0859	0.00293	0.0278	0.00319
δ		0.652	0.115	0.36	0.12

Relation

Table 2 provides a statistical analysis of the overall probability distributions for the relation categories.

Table 2: Statistical analysis of the overall probability distributions for the relation categories. Lengths in millimetres, times in seconds.

Relation	N^k	V	r	x	y
Colleagues	18172	1262 ± 1.7 (σ=234)	840 ± 2.2 (σ=290)	720 ± 1.7 (σ=229)	317 ± 2.6 (σ=344)
Couples	5273	1085 ± 3.2 (σ=231)	699 ± 3.7 (σ=271)	584 ± 2.6 (σ=188)	290 ± 4.4 (σ=318)
Families	12596	1072 ± 2.2 (σ=246)	834 ± 3.2 (σ=357)	592 ± 2.3 (σ=260)	452 ± 4 (σ=447)
Friends	17634	1113 ± 2 (σ=260)	788 ± 2 (σ=265)	659 ± 1.6 (σ=214)	312 ± 2.5 (σ=338)
$F_{3,53671}$		1940	362	975	485
p		$< 10^{-8}$	$< 10^{-8}$	$< 10^{-8}$	$< 10^{-8}$
R^2		0.0978	0.0198	0.0517	0.0264
δ		0.795	0.493	0.614	0.392

Gender

Table 3 provides a statistical analysis of the overall probability distributions for the relation categories.

Age

Table 4 provides a statistical analysis of the overall probability distributions for the minimum age ranges.

Table 3: Statistical analysis of the overall probability distributions for the gender categories. Lengths in millimetres, times in seconds.

Gender	N^k	V	r	x	y
Two females	14688	1075 ± 2.1 (σ=251)	773 ± 2.2 (σ=268)	647 ± 1.7 (σ=202)	302 ± 2.9 (σ=346)
Mixed	19311	1098 ± 1.7 (σ=239)	803 ± 2.4 (σ=334)	614 ± 1.8 (σ=248)	388 ± 3 (σ=411)
Two males	23516	1237 ± 1.6 (σ=249)	839 ± 1.9 (σ=292)	702 ± 1.6 (σ=239)	337 ± 2.3 (σ=355)
$F_{2,57512}$		2570	225	791	232
p		$< 10^{-8}$	$< 10^{-8}$	$< 10^{-8}$	$< 10^{-8}$
R^2		0.0822	0.00778	0.0268	0.008
δ		0.647	0.233	0.365	0.224

Table 4: Statistical analysis of the overall probability distributions for the minimum age ranges. Lengths in millimetres, times in seconds.

Minimum age	N^k	V	r	x	y
0-9 years	1041	1127 ± 8.4 (σ=272)	983 ± 15 (σ=480)	573 ± 9.5 (σ=306)	663 ± 18 (σ=580)
10-19 years	3443	1110 ± 5.2 (σ=303)	767 ± 5.1 (σ=298)	626 ± 3.8 (σ=222)	322 ± 6.2 (σ=364)
20-29 years	18679	1167 ± 1.8 (σ=240)	788 ± 2.1 (σ=289)	665 ± 1.6 (σ=223)	301 ± 2.6 (σ=349)
30-39 years	15552	1179 ± 2.1 (σ=264)	816 ± 2.4 (σ=294)	667 ± 2 (σ=248)	343 ± 2.9 (σ=357)
40-49 years	7974	1167 ± 2.7 (σ=242)	838 ± 3.3 (σ=296)	668 ± 2.7 (σ=243)	374 ± 4.2 (σ=378)
50-59 years	6025	1153 ± 3.3 (σ=253)	812 ± 3.7 (σ=284)	653 ± 2.9 (σ=223)	358 ± 4.7 (σ=367)
60-69 years	3969	1001 ± 3.5 (σ=219)	836 ± 5.4 (σ=340)	643 ± 3.8 (σ=242)	409 ± 6.7 (σ=419)
≥ 70 years	832	877 ± 6 (σ=172)	793 ± 13 (σ=363)	599 ± 7.8 (σ=224)	383 ± 16 (σ=453)
$F_{7,57507}$		400	89.1	46.7	175
p		$< 10^{-8}$	$< 10^{-8}$	$< 10^{-8}$	$< 10^{-8}$
R^2		0.0464	0.0107	0.00566	0.0208
δ		1.16	0.619	0.382	0.991

Height

Table 5 provides a statistical analysis of the overall probability distributions for the minimum height ranges.

Table 5: Statistical analysis of the overall probability distributions for the minimum height ranges. Lengths in millimetres, times in seconds.

Minimum height	N^k	V	r	x	y
< 140 cm	1579	1127 ± 6.9 (σ=274)	942 ± 11 (σ=457)	605 ± 7.6 (σ=300)	578 ± 14 (σ=553)
140-150 cm	2206	1032 ± 6.7 (σ=315)	855 ± 8 (σ=374)	644 ± 5.3 (σ=248)	420 ± 10 (σ=468)
150-160 cm	13064	1076 ± 2.2 (σ=251)	779 ± 2.5 (σ=281)	628 ± 1.8 (σ=209)	337 ± 3.2 (σ=365)
160-170 cm	26345	1151 ± 1.5 (σ=245)	810 ± 1.9 (σ=306)	655 ± 1.5 (σ=243)	348 ± 2.3 (σ=374)
170-180 cm	13497	1234 ± 2.1 (σ=243)	819 ± 2.3 (σ=269)	700 ± 2 (σ=232)	309 ± 2.8 (σ=323)
> 180 cm	824	1224 ± 9.3 (σ=268)	823 ± 11 (σ=325)	686 ± 8.1 (σ=234)	309 ± 14 (σ=404)
$F_{5,57509}$		648	102	149	171
p		$< 10^{-8}$	$< 10^{-8}$	$< 10^{-8}$	$< 10^{-8}$
R^2		0.0533	0.00875	0.0128	0.0146
δ		0.796	0.534	0.398	0.532
