# Supplementary material for: Intrinsic group behaviour: Dependence of pedestrian dyad dynamics on principal social and personal features
Source: PLoS One. 2017 Nov 2;12(11):e0187253. doi: 10.1371/journal.pone.0187253 (PMC5667819; doi:10.1371/journal.pone.0187253)
Supplement: S3 Appendix — (PDF) [file pone.0187253.s012.pdf]

Accounting for other effects

Secondary effects and purpose

Density

Work-oriented dyads are more frequently found during working days, in which the environment presents a lower density (and thus higher velocity and inter-group pedestrian distance[1]). It is thus important to analyse the results regarding purpose in the main text when they are divided for density ranges, for example by comparing results in the $0 \leq \rho < 0.05$ pedestrian per square meter range with those in the $0.15 \leq \rho < 0.2$ range[2]. The results are reported in table 1 and 2, showing that the differences in V and x remain significant regardless of density. The difference in y becomes significant at high density, while at very low density is not significant. The opposite happens for r.

Table 1: Observable dependence on purpose for dyads in the $0 \leq \rho \leq 0.05$ pedestrian per square meter density range. Lengths in millimetres, times in seconds.

Purpose	N_g^k	V	r	x	y
Leisure	426	1113 ± 10 (σ=215)	851 ± 15 (σ=303)	658 ± 9 (σ=186)	400 ± 18 (σ=373)
Work	209	1274 ± 12 (σ=169)	924 ± 20 (σ=296)	741 ± 14 (σ=203)	409 ± 26 (σ=373)
$F_{1,633}$		89.6	8.17	25.7	0.0807
p		$< 10^{-8}$	0.0044	$5.36 \cdot 10^{-7}$	0.776
R^2		0.124	0.0127	0.039	0.000128
δ		0.8	0.242	0.428	0.024

Table 2: Observable dependence on purpose for dyads in the $0.15 \leq \rho \leq 0.2$ pedestrian per square meter density range. Lengths in millimetres, times in seconds.

Purpose	N_g^k	V	r	x	y
Leisure	145	1084 ± 14 (σ=170)	764 ± 17 (σ=209)	560 ± 13 (σ=158)	390 ± 26 (σ=308)
Work	22	1229 ± 27 (σ=125)	754 ± 26 (σ=123)	673 ± 25 (σ=117)	237 ± 40 (σ=186)
$F_{1,165}$		14.7	0.0513	10.2	5.05
p		0.000182	0.821	0.00167	0.026
R^2		0.0817	0.000311	0.0583	0.0297
δ		0.881	0.0521	0.735	0.516

In table 3 and 4 we report, respectively, p and δ values for purpose corresponding to each observable and density range, showing that the V, x and y distributions are different in a statistically significant way at different density ranges, although the effect on y grows with density. Differences in r are significant only at the lowest density range.

[1] F. Zanlungo, D. Brščić and T. Kanda, *Spatial-size scaling of pedestrian groups under growing density conditions* Physical Review E 91 (6), 062810 (2015), citation [2] in the main text.

[2] Groups may contribute to different density ranges, see again citation [2] in the main text for details.

Table 3: p values for purpose corresponding to velocity and distance observables at different density ranges.

Density	V	r	x	y
0-0.05 ped/m^2	$< 10^{-8}$	0.0044	$5.36 \cdot 10^{-7}$	0.776
0.05-0.1 ped/m^2	$< 10^{-8}$	0.682	$< 10^{-8}$	0.000517
0.1-0.15 ped/m^2	$< 10^{-8}$	0.221	$< 10^{-8}$	$1.32 \cdot 10^{-6}$
0.15-0.2 ped/m^2	0.000182	0.821	0.00167	0.026

Table 4: δ values for purpose corresponding to velocity and distance observables at different density ranges.

Density	V	r	x	y
0-0.05 ped/m^2	0.8	0.242	0.428	0.024
0.05-0.1 ped/m^2	0.914	0.0292	0.515	0.248
0.1-0.15 ped/m^2	0.812	0.117	0.627	0.467
0.15-0.2 ped/m^2	0.881	0.0521	0.735	0.516

Gender

The work and leisure populations are strongly biased regarding gender. In tables 5, 6 and 7 we show the results for the work and leisure observables when limited to, respectively, female, mixed and male dyads. While velocity is still significantly different also when gender is fixed, absolute distance in men, group depth in mixed dyads, and all distance observables in females are not significantly different. We may thus conclude that differences between workers and leisure oriented people are present regardless of gender, but are magnified by the gender difference in the two populations.

Table 5: Observable dependence on purpose for 2 female dyads. Lengths in millimetres, times in seconds.

Purpose	N_g^k	V	r	x	y
Leisure	222	1092 ± 13 (σ=194)	794 ± 16 (σ=235)	644 ± 8.4 (σ=125)	328 ± 22 (σ=322)
Work	29	1184 ± 30 (σ=162)	755 ± 28 (σ=150)	663 ± 19 (σ=101)	274 ± 38 (σ=204)
$F_{1,249}$		5.97	0.733	0.615	0.777
p		0.0153	0.393	0.433	0.379
R^2		0.0234	0.00293	0.00247	0.00311
δ		0.484	0.17	0.155	0.175

Age

In tables (8) and (9) we show the results for the work and leisure observables when limited to groups of a given average age. The results suggest that differences may be

Table 6: Observable dependence on purpose for mixed gender dyads. Lengths in millimetres, times in seconds.

Purpose	N_g^k	V	r	x	y
Leisure	330	1097 ± 9.9 (σ=180)	814 ± 15 (σ=267)	602 ± 9.6 (σ=174)	415 ± 19 (σ=341)
Work	41	1226 ± 26 (σ=167)	902 ± 48 (σ=308)	698 ± 24 (σ=152)	420 ± 65 (σ=419)
$F_{1,369}$		18.9	3.79	11.3	0.00662
p		$1.77{\cdot}10^{-5}$	0.0524	0.000849	0.935
R^2		0.0488	0.0102	0.0298	$1.79{\cdot}10^{-5}$
δ		0.722	0.323	0.558	0.0135

Table 7: Observable dependence on purpose for 2 male dyads. Lengths in millimetres, times in seconds.

Purpose	N_g^k	V	r	x	y
Leisure	164	1196 ± 16 (σ=207)	846 ± 19 (σ=246)	660 ± 14 (σ=173)	392 ± 25 (σ=325)
Work	302	1285 ± 8.7 (σ=152)	846 ± 13 (σ=218)	720 ± 9 (σ=157)	325 ± 16 (σ=271)
$F_{1,464}$		28.1	0.000251	14.4	5.5
p		$1.83{\cdot}10^{-7}$	0.987	0.000165	0.0195
R^2		0.057	$5.42{\cdot}10^{-7}$	0.0301	0.0117
δ		0.515	0.00154	0.369	0.228

present at any age (in particular concerning V), but are definitely more strong for more mature walkers.

Table 8: Observable dependence on purpose for dyads with average age in the 20-29 years range. Lengths in millimetres, times in seconds.

Purpose	N_g^k	V	r	x	y
Leisure	292	1164 ± 10 (σ=177)	798 ± 14 (σ=242)	656 ± 9.7 (σ=165)	326 ± 17 (σ=290)
Work	78	1242 ± 19 (σ=166)	775 ± 17 (σ=152)	684 ± 12 (σ=108)	266 ± 22 (σ=197)
$F_{1,368}$		12.2	0.608	1.91	2.93
p		0.000536	0.436	0.168	0.088
R^2		0.0321	0.00165	0.00515	0.00789
δ		0.446	0.0996	0.176	0.219

In table 10 and 11 we report, respectively, p and δ values for purpose corresponding to each observable and average age range, showing again that differences have a tendency to be more pronounced in the high age ranges.

Height

In tables (12) and (13) we show the results for the work and leisure observables when limited to groups of a given average height, and in tables 14 and 15 we report, respectively, p and δ values for purpose corresponding to each observable and average height

Table 9: Observable dependence on purpose for dyads with average age in the 50-59 years range. Lengths in millimetres, times in seconds.

Purpose	N_g^k	V	r	x	y
Leisure	61	1053 ± 21 (σ=164)	808 ± 32 (σ=247)	601 ± 20 (σ=155)	404 ± 46 (σ=356)
Work	53	1276 ± 21 (σ=153)	845 ± 24 (σ=173)	706 ± 20 (σ=144)	345 ± 36 (σ=261)
$F_{1,112}$		54.8	0.808	13.7	0.966
p		$< 10^{-8}$	0.371	0.000328	0.328
R^2		0.329	0.00716	0.109	0.00855
δ		1.4	0.17	0.702	0.186

Table 10: p values for purpose corresponding to velocity and distance observables at different average age ranges.

Average age	V	r	x	y
20-29 years	0.000536	0.436	0.168	0.088
30-39 years	$< 10^{-8}$	0.0689	$6.34 \cdot 10^{-8}$	0.144
40-49 years	$< 10^{-8}$	0.12	$4.78 \cdot 10^{-6}$	0.264
50-59 years	$< 10^{-8}$	0.371	0.000328	0.328
60-69 years	0.0233	0.221	0.48	0.463

Table 11: δ values for purpose corresponding to velocity and distance observables at different average age ranges.

Average age	V	r	x	y
20-29 years	0.446	0.0996	0.176	0.219
30-39 years	0.994	0.224	0.682	0.18
40-49 years	0.97	0.226	0.68	0.162
50-59 years	1.4	0.17	0.702	0.186
60-69 years	1.21	0.649	0.373	0.389

range. Differences appear to be more pronounced for higher height, probably affected also by the gender distributions.

Table 12: Observable dependence on purpose for dyads with average height in the 150-160 cm range. Lengths in millimetres, times in seconds.

Purpose	N_g^k	V	r	x	y
Leisure	108	1107 ± 25 (σ=260)	821 ± 26 (σ=268)	629 ± 15 (σ=153)	389 ± 34 (σ=352)
Work	10	1152 ± 51 (σ=160)	709 ± 27 (σ=86)	631 ± 28 (σ=88.5)	264 ± 32 (σ=101)
$F_{1,116}$		0.283	1.71	0.00249	1.24
p		0.596	0.194	0.96	0.268
R^2		0.00243	0.0145	$2.15 \cdot 10^{-5}$	0.0106
δ		0.177	0.434	0.0166	0.37

Table 13: Observable dependence on purpose for dyads with average age in the 170-180 cm range. Lengths in millimetres, times in seconds.

Purpose	N_g^k	V	r	x	y
Leisure	188	1138 ± 12 (σ=168)	801 ± 15 (σ=212)	628 ± 12 (σ=159)	366 ± 21 (σ=291)
Work	233	1291 ± 10 (σ=157)	850 ± 15 (σ=230)	730 ± 10 (σ=153)	322 ± 18 (σ=273)
$F_{1,419}$		92.2	5.22	44	2.53
p		$< 10^{-8}$	0.0228	$< 10^{-8}$	0.113
R^2		0.18	0.0123	0.0951	0.006
δ		0.944	0.225	0.652	0.156

Table 14: p values for purpose corresponding to velocity and distance observables at different average age ranges.

Average height	V	r	x	y
150-160 cm	0.596	0.194	0.96	0.268
160-170 cm	$< 10^{-8}$	0.0557	0.00126	0.953
170-180 cm	$< 10^{-8}$	0.0228	$< 10^{-8}$	0.113
$>$ 180 cm	0.773	0.959	0.289	0.522

Table 15: δ values for purpose corresponding to velocity and distance observables at different average age ranges.

Average height	V	r	x	y
150-160 cm	0.177	0.434	0.0166	0.37
160-170 cm	0.696	0.217	0.368	0.00667
170-180 cm	0.944	0.225	0.652	0.156
$>$ 180 cm	0.0998	0.0177	0.368	0.22

Secondary effects and relation

Density

As discussed above, work-oriented (and thus colleagues) dyads are more present during working days, in which the environment presents a lower density. Tables 16 and 17 show the observables dependence for fixed density ranges ($0 \leq \rho < 0.05$ ped/m^2 and $0.15 \leq \rho < 0.2$ ped/m^2, respectively). The major trends exposed in the main text are present at any density, as confirmed also by tables 18 and 19, reporting p and δ values, respectively, for all density ranges.

Table 16: Observable dependence on relation for dyads in the $0 \leq \rho < 0.05$ ped/m^2 range. Lengths in millimetres, times in seconds.

Relation	N_g^k	V	r	x	y
Colleagues	202	1276 ± 12 (σ=169)	934 ± 21 (σ=298)	751 ± 15 (σ=207)	409 ± 26 (σ=376)
Couples	62	1103 ± 25 (σ=193)	760 ± 38 (σ=297)	600 ± 22 (σ=177)	359 ± 40 (σ=314)
Families	125	1084 ± 19 (σ=208)	894 ± 30 (σ=331)	617 ± 16 (σ=175)	512 ± 37 (σ=413)
Friends	193	1130 ± 17 (σ=230)	830 ± 19 (σ=258)	685 ± 12 (σ=162)	338 ± 23 (σ=326)
$F_{3,578}$		30.5	7.59	19	6.17
p		$< 10^{-8}$	$5.52 \cdot 10^{-5}$	$< 10^{-8}$	0.000396
R^2		0.137	0.0379	0.0896	0.031
δ		1.03	0.585	0.754	0.482

Table 17: Observable dependence on relation for dyads in the $0.15 \leq \rho < 0.2$ ped/m^2 range. Lengths in millimetres, times in seconds.

Relation	N_g^k	V	r	x	y
Colleagues	22	1229 ± 27 (σ=125)	754 ± 26 (σ=123)	673 ± 25 (σ=117)	237 ± 40 (σ=186)
Couples	19	1064 ± 28 (σ=124)	663 ± 31 (σ=135)	542 ± 22 (σ=97.1)	290 ± 36 (σ=159)
Families	68	1068 ± 22 (σ=180)	802 ± 30 (σ=247)	532 ± 21 (σ=170)	465 ± 44 (σ=362)
Friends	57	1107 ± 22 (σ=168)	753 ± 22 (σ=164)	603 ± 20 (σ=149)	332 ± 33 (σ=251)
$F_{3,162}$		5.54	2.59	5.87	4.77
p		0.0012	0.055	0.000794	0.00327
R^2		0.0931	0.0457	0.098	0.0811
δ		1.32	0.613	0.888	0.694

Gender

We now compare the results regarding relation for groups of given gender (two females, mixed and two males) in tables 20, 21 and 22. Differences in the distributions (and the corresponding trends) are still significant in fixed gender groups, with the exception of the r female and male distributions, although, as shown by the relatively high δ values, this may be due due by the low amount of data in some categories. The patterns analysed in the main text are mostly respected, although we may notice some differences such

Table 18: p values for relation corresponding to velocity and distance observables at different density ranges.

Density	V	r	x	y
0-0.05 ped/m^2	$< 10^{-8}$	$5.52 \cdot 10^{-5}$	$< 10^{-8}$	0.000396
0.05-0.1 ped/m^2	$< 10^{-8}$	0.000158	$< 10^{-8}$	$< 10^{-8}$
0.1-0.15 ped/m^2	$< 10^{-8}$	$2.22 \cdot 10^{-5}$	$< 10^{-8}$	$< 10^{-8}$
0.15-0.2 ped/m^2	0.0012	0.055	0.000794	0.00327
0.2-0.25 ped/m^2	0.378	0.327	0.144	0.664

Table 19: δ values for relation corresponding to velocity and distance observables at different density ranges.

Density	V	r	x	y
0-0.05 ped/m^2	1.03	0.585	0.754	0.482
0.05-0.1 ped/m^2	1.07	0.476	0.732	0.538
0.1-0.15 ped/m^2	1.16	0.666	0.857	0.761
0.15-0.2 ped/m^2	1.32	0.613	0.888	0.694
0.2-0.25 ped/m^2	0.675	0.798	0.983	0.974

as female friends walking at an higher distance than colleagues, and two male families walking at a very high speed.

Table 20: Observable dependence on relation for 2 females dyads. Lengths in millimetres, times in seconds.

Relation	N_g^k	V	r	x	y
Colleagues	24	1167 ± 30 (σ=145)	735 ± 26 (σ=128)	664 ± 20 (σ=95.9)	238 ± 34 (σ=168)
Families	28	1023 ± 32 (σ=171)	847 ± 58 (σ=305)	565 ± 27 (σ=140)	488 ± 77 (σ=405)
Friends	184	1105 ± 15 (σ=197)	777 ± 15 (σ=205)	658 ± 8.5 (σ=115)	293 ± 20 (σ=274)
$F_{2,233}$		3.85	1.9	7.85	6.48
p		0.0227	0.153	0.000503	0.00182
R^2		0.032	0.016	0.0631	0.0527
δ		0.902	0.465	0.817	0.783

Age

Tables 23 and 24 show the observables dependence for fixed minimum age ranges (20-29 and 50-59 years, respectively). The major trends exposed in the main text are present even when the age is kept fixed. We may notice that y assumes a very high value in families even when children are not present[3]. p and δ values for the relation feature at different minimum age ranges are shown in, respectively, tables 25 and 26.

[3] This may be related to a selection bias in coders, that may have labelled mixed gender dyads as "couples" or "families" depending on their proximity and "abreastness".

Table 21: Observable dependence on relation for mixed gender dyads. Lengths in millimetres, times in seconds.

Relation	N_g^k	V	r	x	y
Colleagues	35	1228 ± 30 (σ=175)	923 ± 55 (σ=327)	702 ± 27 (σ=158)	440 ± 75 (σ=445)
Couples	96	1099 ± 17 (σ=169)	714 ± 22 (σ=219)	600 ± 15 (σ=150)	291 ± 24 (σ=231)
Families	183	1078 ± 13 (σ=182)	860 ± 21 (σ=285)	588 ± 13 (σ=173)	493 ± 28 (σ=372)
Friends	20	1153 ± 41 (σ=183)	820 ± 43 (σ=192)	616 ± 43 (σ=192)	391 ± 70 (σ=311)
$F_{3,330}$		7.47	7.99	4.63	7.26
p		$7.49 \cdot 10^{-5}$	$3.72 \cdot 10^{-5}$	0.00345	$9.96 \cdot 10^{-5}$
R^2		0.0636	0.0677	0.0404	0.0619
δ		0.832	0.83	0.67	0.61

Table 22: Observable dependence on relation for 2 males dyads. Lengths in millimetres, times in seconds.

Relation	N_g^k	V	r	x	y
Colleagues	299	1287 ± 8.8 (σ=152)	852 ± 13 (σ=220)	724 ± 9.3 (σ=160)	329 ± 16 (σ=273)
Families	35	1234 ± 39 (σ=229)	891 ± 63 (σ=375)	571 ± 31 (σ=182)	537 ± 79 (σ=467)
Friends	114	1187 ± 19 (σ=198)	811 ± 17 (σ=186)	676 ± 14 (σ=147)	335 ± 23 (σ=246)
$F_{2,445}$		14.5	2.1	16.1	8.35
p		$8.13 \cdot 10^{-7}$	0.124	$1.76 \cdot 10^{-7}$	0.000276
R^2		0.0611	0.00933	0.0675	0.0362
δ		0.607	0.329	0.94	0.698

Table 23: Observable dependence on relation for dyads with minimum age in the 20-29 years range. Lengths in millimetres, times in seconds.

Relation	N_g^k	V	r	x	y
Colleagues	86	1255 ± 18 (σ=165)	813 ± 20 (σ=185)	706 ± 16 (σ=144)	291 ± 24 (σ=219)
Couples	74	1115 ± 19 (σ=165)	711 ± 27 (σ=229)	600 ± 18 (σ=154)	281 ± 28 (σ=243)
Families	23	1109 ± 39 (σ=187)	877 ± 58 (σ=277)	581 ± 37 (σ=177)	527 ± 78 (σ=373)
Friends	164	1186 ± 13 (σ=164)	801 ± 16 (σ=208)	683 ± 10 (σ=128)	298 ± 21 (σ=265)
$F_{3,343}$		10.9	5.05	11.1	5.9
p		$7.21 \cdot 10^{-7}$	0.00195	$5.89 \cdot 10^{-7}$	0.00062
R^2		0.0872	0.0423	0.0883	0.0491
δ		0.861	0.684	0.825	0.882

Height

Tables 27 and 28 show the observables dependence for fixed average height ranges (160-170 and 170-180 cm, respectively), showing that the distributions are still different in a significant way, and that the major patterns exposed in the main text are confirmed. Tables 29 and 30 show respectively the p and δ values for all height ranges. δ values are always high showing that some reduced p values are due to the low number of groups in some ranges.

Table 24: Observable dependence on relation for dyads with minimum age in the 50-59 years range. Lengths in millimetres, times in seconds.

Relation	N_g^k	V	r	x	y
Colleagues	52	1274 ± 22 (σ=159)	844 ± 24 (σ=172)	700 ± 20 (σ=142)	350 ± 36 (σ=263)
Families	28	1048 ± 32 (σ=169)	846 ± 55 (σ=289)	562 ± 34 (σ=182)	492 ± 78 (σ=410)
Friends	22	1051 ± 36 (σ=167)	759 ± 44 (σ=208)	637 ± 24 (σ=115)	308 ± 59 (σ=276)
$F_{2,99}$		23.4	1.29	7.63	2.55
p		$< 10^{-8}$	0.28	0.000825	0.0835
R^2		0.321	0.0254	0.134	0.0489
δ		1.39	0.339	0.878	0.514

Table 25: p values for relation in different minimum age ranges.

Minimum age	V	r	x	y
10-19 years	0.558	0.049	0.615	0.0313
20-29 years	$7.21 \cdot 10^{-7}$	0.00195	$5.89 \cdot 10^{-7}$	0.00062
30-39 years	$< 10^{-8}$	0.0128	$< 10^{-8}$	0.0513
40-49 years	$1.02 \cdot 10^{-5}$	0.39	0.000266	0.537
50-59 years	$< 10^{-8}$	0.28	0.000825	0.0835
60-69 years	0.0525	0.248	0.745	0.388
$\geq$ 70 years	0.385	0.251	0.198	0.237

Table 26: δ values for relation in different minimum age ranges.

Minimum age	V	r	x	y
10-19 years	0.888	3.23	0.609	2.49
20-29 years	0.861	0.684	0.825	0.882
30-39 years	1.48	0.77	1.05	0.636
40-49 years	1.2	0.529	0.848	0.557
50-59 years	1.39	0.339	0.878	0.514
60-69 years	1.21	1.12	0.386	0.669
$\geq$ 70 years	0.612	0.844	0.937	0.865

Table 27: Observable dependence on relation for dyads with average height in the 160-170 cm range. Lengths in millimetres, times in seconds.

Relation	N_g^k	V	r	x	y
Colleagues	89	1240 ± 16 (σ=149)	862 ± 26 (σ=249)	686 ± 18 (σ=167)	380 ± 37 (σ=350)
Couples	47	1106 ± 28 (σ=191)	731 ± 33 (σ=226)	622 ± 28 (σ=189)	287 ± 30 (σ=204)
Families	121	1090 ± 18 (σ=196)	854 ± 27 (σ=295)	593 ± 15 (σ=169)	487 ± 34 (σ=371)
Friends	172	1135 ± 13 (σ=169)	798 ± 17 (σ=221)	659 ± 10 (σ=131)	321 ± 23 (σ=302)
$F_{3,425}$		13.3	3.96	7.08	7.42
p		$2.47 \cdot 10^{-8}$	0.0084	0.000119	$7.42 \cdot 10^{-5}$
R^2		0.086	0.0272	0.0476	0.0498
δ		0.843	0.542	0.551	0.599

Table 28: Observable dependence on relation for dyads with average age in the 170-180 cm range. Lengths in millimetres, times in seconds.

Relation	N_g^k	V	r	x	y
Colleagues	231	1293 ± 10 (σ=157)	859 ± 15 (σ=232)	738 ± 10 (σ=157)	325 ± 18 (σ=274)
Couples	45	1089 ± 22 (σ=145)	700 ± 33 (σ=219)	576 ± 14 (σ=95.4)	300 ± 39 (σ=264)
Families	56	1107 ± 22 (σ=166)	818 ± 31 (σ=234)	557 ± 20 (σ=148)	462 ± 48 (σ=361)
Friends	71	1162 ± 20 (σ=166)	811 ± 19 (σ=156)	679 ± 17 (σ=145)	328 ± 26 (σ=215)
$F_{3,399}$		38.9	6.77	31.5	4.13
p		$< 10^{-8}$	0.000183	$< 10^{-8}$	0.00672
R^2		0.226	0.0485	0.192	0.0301
δ		1.32	0.692	1.16	0.503

Table 29: p values for relation at different average height ranges.

Average height	V	r	x	y
< 140 cm	0.362	0.108	0.61	0.0849
140-150 cm	0.12	0.181	0.785	0.299
150-160 cm	0.842	0.133	0.803	0.0402
160-170 cm	$2.47 \cdot 10^{-8}$	0.0084	0.000119	$7.42 \cdot 10^{-5}$
170-180 cm	$< 10^{-8}$	0.000183	$< 10^{-8}$	0.00672
> 180 cm	0.00432	0.551	0.126	0.951

Table 30: δ values for relation at different average height ranges.

Average height	V	r	x	y
< 140 cm	0.767	1.41	0.423	1.53
140-150 cm	0.977	0.798	0.159	0.612
150-160 cm	0.405	0.658	0.521	0.594
160-170 cm	0.843	0.542	0.551	0.599
170-180 cm	1.32	0.692	1.16	0.503
> 180 cm	2.78	0.972	1.41	0.331

Secondary effects and gender

Density

Tables 31 and 32 show the dependence on gender of observables at fixed density ranges ($0 \leq \rho < 0.05$ ped/m^2 and $0.15 \leq \rho < 0.2$ ped/m^2, respectively). We may see that only the r observable loses the statistically significant gender dependence at high density (but still shows it at lower density, when pedestrians may move more freely; furthermore, the effect size is almost not affected by density), while the other observables show it at low and high density. Tables 33 and 34 show the dependence of, respectively, the gender p and δ values at different density values.

Table 31: Observable dependence on gender in the $0 \leq \rho < 0.05$ ped/m^2 density range. Lengths in millimetres, times in seconds.

Gender	N_g^k	V	r	x	y
Two females	160	1095 ± 17 (σ=219)	818 ± 21 (σ=267)	669 ± 11 (σ=138)	337 ± 27 (σ=346)
Mixed	217	1112 ± 13 (σ=196)	870 ± 23 (σ=340)	642 ± 13 (σ=194)	448 ± 28 (σ=409)
Two males	259	1254 ± 12 (σ=196)	914 ± 18 (σ=283)	733 ± 13 (σ=217)	404 ± 22 (σ=351)
$F_{2,633}$		41.5	5.06	14.1	4.11
p		$< 10^{-8}$	0.00658	$1.04 \cdot 10^{-6}$	0.0169
R^2		0.116	0.0157	0.0426	0.0128
δ		0.771	0.346	0.441	0.289

Table 32: Observable dependence on gender in the $0.15 \leq \rho < 0.2$ ped/m^2 density range. Lengths in millimetres, times in seconds.

Gender	N_g^k	V	r	x	y
Two females	35	1073 ± 28 (σ=164)	714 ± 26 (σ=152)	572 ± 18 (σ=107)	318 ± 39 (σ=230)
Mixed	73	1062 ± 18 (σ=152)	782 ± 29 (σ=247)	521 ± 20 (σ=172)	448 ± 42 (σ=361)
Two males	59	1171 ± 23 (σ=178)	767 ± 19 (σ=147)	644 ± 18 (σ=136)	304 ± 28 (σ=218)
$F_{2,164}$		7.81	1.4	11.2	4.6
p		0.000578	0.249	$2.88 \cdot 10^{-5}$	0.0114
R^2		0.0869	0.0168	0.12	0.0531
δ		0.665	0.308	0.786	0.471

Table 33: p values for gender in different density ranges.

Density	V	r	x	y
0-0.05 ped/m^2	$< 10^{-8}$	0.00658	$1.04 \cdot 10^{-6}$	0.0169
0.05-0.1 ped/m^2	$< 10^{-8}$	0.0448	$< 10^{-8}$	0.00164
0.1-0.15 ped/m^2	$< 10^{-8}$	0.897	$9.41 \cdot 10^{-8}$	0.00478
0.15-0.2 ped/m^2	0.000578	0.249	$2.88 \cdot 10^{-5}$	0.0114
0.2-0.25 ped/m^2	0.0304	0.0628	0.31	0.43

Table 34: δ values for gender in different density ranges.

Density	V	r	x	y
0-0.05 ped/m^2	0.771	0.346	0.441	0.289
0.05-0.1 ped/m^2	0.771	0.235	0.537	0.291
0.1-0.15 ped/m^2	0.737	0.0554	0.582	0.35
0.15-0.2 ped/m^2	0.665	0.308	0.786	0.471
0.2-0.25 ped/m^2	1.31	1.56	0.942	0.751

Relation

Tables 35, 36 and 37 show the gender dependence of observables in, respectively, colleagues, families and friends (couples are not shown being exclusively of mixed gender).

Table 35: Observable dependence on gender for colleagues. Lengths in millimetres, times in seconds.

Gender	N_g^k	V	r	x	y
Two females	24	1167 ± 30 (σ=145)	735 ± 26 (σ=128)	664 ± 20 (σ=95.9)	238 ± 34 (σ=168)
Mixed	35	1228 ± 30 (σ=175)	923 ± 55 (σ=327)	702 ± 27 (σ=158)	440 ± 75 (σ=445)
Two males	299	1287 ± 8.8 (σ=152)	852 ± 13 (σ=220)	724 ± 9.3 (σ=160)	329 ± 16 (σ=273)
$F_{2,355}$		8.49	4.82	1.78	3.7
p		0.00025	0.00862	0.17	0.0256
R^2		0.0457	0.0264	0.00995	0.0204
δ		0.798	0.709	0.38	0.561

Table 36: Observable dependence on gender for families. Lengths in millimetres, times in seconds.

Gender	N_g^k	V	r	x	y
Two females	28	1023 ± 32 (σ=171)	847 ± 58 (σ=305)	565 ± 27 (σ=140)	488 ± 77 (σ=405)
Mixed	183	1078 ± 13 (σ=182)	860 ± 21 (σ=285)	588 ± 13 (σ=173)	493 ± 28 (σ=372)
Two males	35	1234 ± 39 (σ=229)	891 ± 63 (σ=375)	571 ± 31 (σ=182)	537 ± 79 (σ=467)
$F_{2,243}$		12.3	0.197	0.308	0.198
p		$8.41\cdot10^{-6}$	0.821	0.735	0.821
R^2		0.0917	0.00162	0.00253	0.00163
δ		1.03	0.128	0.135	0.112

We may see that males dyads are farther and faster than female ones regardless of relation (although the differences in r, x and y are quite reduced in families and friends). Mixed dyad behaviour, on the other hand, depends strongly on relation. Mixed dyads are the only ones including couples, and this affects strongly their behaviour, and represent also the largest part of families. They are very little represented in friends (interestingly, mixed dyads of friends walk much closer, in abreast distance, than same sex dyads, although their absolute distance is higher than in females). The "colleagues"

Table 37: Observable dependence on gender for friends. Lengths in millimetres, times in seconds.

Gender	N_g^k	V	r	x	y
Two females	184	1105 ± 15 (σ=197)	777 ± 15 (σ=205)	658 ± 8.5 (σ=115)	293 ± 20 (σ=274)
Mixed	20	1153 ± 41 (σ=183)	820 ± 43 (σ=192)	616 ± 43 (σ=192)	391 ± 70 (σ=311)
Two males	114	1187 ± 19 (σ=198)	811 ± 17 (σ=186)	676 ± 14 (σ=147)	335 ± 23 (σ=246)
$F_{2,315}$		6.02	1.27	1.91	1.76
p		0.00272	0.283	0.15	0.173
R^2		0.0368	0.00798	0.012	0.0111
δ		0.412	0.213	0.388	0.354

category could represent a fair field for comparing the effect of gender, and in it the mixed behaviour is somehow in between the two sexes (although the absolute distance r and group depth y are very large, suggesting not abreast formations) but in our set colleagues are extremely biased towards males, and thus the analysis in hindered by low female and mixed dyads numbers. Finally we may notice that in families and friends, the effect of gender on distance (r, x and y) is very reduced, but the one on velocity is persistent. The velocity effect size in families is nevertheless more than two times the one for friends.

Age

Tables 38 and 39 show the dependence on gender of observables at fixed average age ranges (20-29 years and 50-59 years, respectively). Interestingly, the differences between young two females and two males dyads are reduced (and almost absent regarding the distance observables r, x and y), while they are very strong in elder groups. Young mixed dyad behaviour is strongly influenced by the presence of couples.

Table 38: Observable dependence on gender in the 20-29 years average age range. Lengths in millimetres, times in seconds.

Gender	N_g^k	V	r	x	y
Two females	111	1166 ± 16 (σ=170)	791 ± 21 (σ=220)	686 ± 10 (σ=110)	275 ± 26 (σ=271)
Mixed	125	1122 ± 16 (σ=175)	784 ± 23 (σ=255)	612 ± 16 (σ=182)	360 ± 27 (σ=307)
Two males	134	1247 ± 14 (σ=164)	803 ± 17 (σ=201)	689 ± 13 (σ=148)	301 ± 20 (σ=235)
$F_{2,367}$		18	0.235	10.5	3.03
p		$3.37 \cdot 10^{-8}$	0.791	$3.77 \cdot 10^{-5}$	0.0496
R^2		0.0895	0.00128	0.054	0.0162
δ		0.739	0.0832	0.47	0.291

Tables 40 and 41 show the p and δ values for gender in different average age ranges. Minimum ages ranges are shown in tables 42 and 43.

Table 39: Observable dependence on gender in the 50-59 years average age range. Lengths in millimetres, times in seconds.

Gender	N_g^k	V	r	x	y
Two females	20	1010 ± 30 (σ=136)	708 ± 21 (σ=95.5)	613 ± 27 (σ=121)	254 ± 34 (σ=151)
Mixed	34	1071 ± 29 (σ=170)	856 ± 48 (σ=278)	608 ± 32 (σ=189)	462 ± 66 (σ=388)
Two males	60	1255 ± 22 (σ=168)	847 ± 25 (σ=192)	686 ± 18 (σ=141)	369 ± 38 (σ=298)
$F_{2,111}$		22.8	3.69	3.37	2.81
p		$< 10^{-8}$	0.0281	0.0379	0.0643
R^2		0.291	0.0623	0.0573	0.0482
δ		1.52	0.646	0.486	0.646

Table 40: p values for gender in different average age ranges.

Average age	V	r	x	y
10-19 years	0.0301	0.685	0.573	0.903
20-29 years	$3.37{\cdot}10^{-8}$	0.791	$3.77{\cdot}10^{-5}$	0.0496
30-39 years	$< 10^{-8}$	0.0477	$7.66{\cdot}10^{-8}$	0.0433
40-49 years	$< 10^{-8}$	0.106	0.000167	0.856
50-59 years	$< 10^{-8}$	0.0281	0.0379	0.0643
60-69 years	0.00145	0.495	0.17	0.655
$\geq$ 70 years	0.245	0.564	0.543	0.598

Table 41: δ values for gender in different average age ranges.

Average age	V	r	x	y
10-19 years	0.769	0.241	0.309	0.14
20-29 years	0.739	0.0832	0.47	0.291
30-39 years	0.87	0.48	0.732	0.322
40-49 years	1.3	0.329	0.619	0.0946
50-59 years	1.52	0.646	0.486	0.646
60-69 years	1.49	0.457	0.666	0.27
$\geq$ 70 years	1.56	0.802	0.915	0.788

Table 42: p values for gender in different minimum age ranges.

Minimum age	V	r	x	y
0-9 years	0.0872	0.17	0.577	0.198
10-19 years	0.00563	0.497	0.981	0.484
20-29 years	$1.67{\cdot}10^{-7}$	0.665	$8.91{\cdot}10^{-5}$	0.0654
30-39 years	$< 10^{-8}$	0.0904	$1.99{\cdot}10^{-8}$	0.027
40-49 years	$3.02{\cdot}10^{-8}$	0.193	0.000602	0.778
50-59 years	$3.78{\cdot}10^{-8}$	0.0458	0.0555	0.0743
60-69 years	0.00245	0.446	0.105	0.651
$\geq$ 70 years	0.245	0.564	0.543	0.598

Table 43: δ values for gender in different minimum age ranges.

Minimum age	V	r	x	y
0-9 years	1.11	1.21	0.449	1.17
10-19 years	0.949	0.473	0.0487	0.421
20-29 years	0.715	0.111	0.475	0.271
30-39 years	0.907	0.396	0.749	0.306
40-49 years	1.62	0.343	0.65	0.113
50-59 years	1.47	0.616	0.487	0.659
60-69 years	1.45	0.541	0.79	0.275
$\geq$ 70 years	1.56	0.802	0.915	0.788

Height

Tables 44 and 45 show the dependence on gender of observables at fixed average height ranges (150-160 and 170-180 cm, respectively). The results (in particular for the shorter height range the effect size, that helps in dealing with the reduced number of groups) show that differences between the sexes are still present when we consider individuals of similar height.

Table 44: Observable dependence on gender in the 150-160 cm average height range. Lengths in millimetres, times in seconds.

Gender	N_g^k	V	r	x	y
Two females	75	1094 $\pm$ 27 (σ=232)	791 $\pm$ 26 (σ=225)	627 $\pm$ 15 (σ=131)	352 $\pm$ 36 (σ=316)
Mixed	25	1045 $\pm$ 35 (σ=176)	796 $\pm$ 43 (σ=217)	603 $\pm$ 33 (σ=167)	376 $\pm$ 60 (σ=300)
Two males	18	1272 $\pm$ 82 (σ=346)	921 $\pm$ 92 (σ=390)	674 $\pm$ 42 (σ=180)	493 $\pm$ 110 (σ=447)
$F_{2,115}$		4.95	1.89	1.22	1.24
p		0.00869	0.156	0.3	0.294
R^2		0.0792	0.0318	0.0207	0.0211
δ		0.873	0.493	0.415	0.408

Table 45: Observable dependence on gender in the 170-180 cm average height range. Lengths in millimetres, times in seconds.

Gender	N_g^k	V	r	x	y
Two females	16	1091 $\pm$ 48 (σ=191)	741 $\pm$ 30 (σ=119)	662 $\pm$ 22 (σ=88.5)	238 $\pm$ 32 (σ=128)
Mixed	121	1127 $\pm$ 16 (σ=171)	797 $\pm$ 23 (σ=250)	598 $\pm$ 14 (σ=149)	396 $\pm$ 31 (σ=338)
Two males	284	1270 $\pm$ 9.6 (σ=161)	846 $\pm$ 13 (σ=213)	723 $\pm$ 9.4 (σ=159)	324 $\pm$ 15 (σ=258)
$F_{2,418}$		36.7	3.4	27.8	3.89
p		$< 10^{-8}$	0.0344	$< 10^{-8}$	0.0212
R^2		0.149	0.016	0.117	0.0183
δ		1.1	0.502	0.799	0.492

Tables 46 and 47 show, respectively, the gender p and δ values for different average height ranges.

Table 46: Gender p values for different average height ranges.

Average height	V	r	x	y
$<$ 140 cm	0.614	0.0596	0.958	0.148
140-150 cm	0.000737	0.372	0.0226	0.306
150-160 cm	0.00869	0.156	0.3	0.294
160-170 cm	$1.0 \cdot 10^{-5}$	0.0653	0.0212	0.000455
170-180 cm	$< 10^{-8}$	0.0344	$< 10^{-8}$	0.0212
$>$ 180 cm	0.0241	0.191	0.137	0.647

Table 47: Gender δ values for different average height ranges.

Average height	V	r	x	y
$<$ 140 cm	0.774	1.89	0.183	1.39
140-150 cm	2.06	0.693	1.47	0.868
150-160 cm	0.873	0.493	0.415	0.408
160-170 cm	0.523	0.279	0.235	0.416
170-180 cm	1.1	0.502	0.799	0.492
$>$ 180 cm	1.28	0.708	0.811	0.246

Secondary effects and age

Density

Tables 48 and 49 show the age dependence of observables in, respectively, the $0 \le \rho \le 0.05$ ped/m^2 and $0.15 \le \rho \le 0.2$ ped/m^2 density ranges. Results mostly reflect those of the main text, with high or relatively high δ values suggesting that some not very good p values may be due to the scarcity of data in the children and elderly categories (i.e. the categories with the most different behaviour). A remarkable feature, presented with the caveats related to sensor noise in the tracking of children, is that while in general velocity decreases with density, this is not true for dyads with children, as shown in the supplementary figure S3.

Table 48: Observable dependence on minimum age in the $0 \le \rho \le 0.05$ ped/m^2 density range. Lengths in millimetres, times in seconds.

Minimum age	N_g^k	V	r	x	y
0-9 years	9	1075 ± 68 (σ=205)	1078 ± 97 (σ=291)	663 ± 62 (σ=186)	704 ± 140 (σ=414)
10-19 years	44	1175 ± 43 (σ=288)	802 ± 40 (σ=262)	661 ± 25 (σ=167)	337 ± 44 (σ=294)
20-29 years	184	1198 ± 14 (σ=196)	853 ± 23 (σ=313)	694 ± 14 (σ=193)	357 ± 27 (σ=372)
30-39 years	185	1196 ± 16 (σ=217)	894 ± 22 (σ=306)	696 ± 16 (σ=223)	418 ± 27 (σ=368)
40-49 years	87	1150 ± 20 (σ=191)	909 ± 32 (σ=297)	683 ± 22 (σ=202)	440 ± 42 (σ=395)
50-59 years	71	1157 ± 23 (σ=198)	844 ± 27 (σ=228)	678 ± 18 (σ=149)	381 ± 38 (σ=320)
60-69 years	47	1022 ± 25 (σ=174)	912 ± 51 (σ=348)	670 ± 27 (σ=182)	481 ± 62 (σ=424)
≥ 70 years	9	891 ± 31 (σ=92.6)	815 ± 100 (σ=307)	605 ± 19 (σ=55.8)	411 ± 130 (σ=394)
$F_{7,628}$		6.98	1.61	0.527	1.94
p		$4.97 \cdot 10^{-8}$	0.129	0.815	0.0613
R^2		0.0722	0.0176	0.00584	0.0211
δ		1.59	1.03	0.419	1.16

Table 49: Observable dependence on minimum age in the $0.15 \le \rho \le 0.2$ ped/m^2 density range. Lengths in millimetres, times in seconds.

Minimum age	N_g^k	V	r	x	y
0-9 years	6	1284 ± 110 (σ=258)	693 ± 51 (σ=126)	485 ± 47 (σ=116)	401 ± 92 (σ=225)
10-19 years	14	1146 ± 47 (σ=176)	806 ± 65 (σ=244)	571 ± 39 (σ=147)	426 ± 91 (σ=341)
20-29 years	72	1099 ± 16 (σ=133)	745 ± 20 (σ=167)	598 ± 17 (σ=145)	322 ± 30 (σ=255)
30-39 years	39	1102 ± 31 (σ=192)	766 ± 33 (σ=208)	575 ± 24 (σ=149)	372 ± 50 (σ=313)
40-49 years	17	1121 ± 32 (σ=131)	763 ± 37 (σ=152)	547 ± 42 (σ=172)	403 ± 68 (σ=279)
50-59 years	10	1057 ± 53 (σ=167)	739 ± 60 (σ=190)	485 ± 58 (σ=184)	416 ± 110 (σ=343)
60-69 years	7	1021 ± 75 (σ=199)	967 ± 130 (σ=354)	616 ± 84 (σ=222)	618 ± 160 (σ=423)
≥ 70 years	2	760 ± 18 (σ=25.4)	644 ± 22 (σ=31)	585 ± 8.9 (σ=12.5)	185 ± 52 (σ=73.5)
$F_{7,159}$		2.76	1.46	1.11	1.21
p		0.00993	0.184	0.359	0.299
R^2		0.108	0.0606	0.0466	0.0506
δ		2.22	0.987	0.652	1.1

Tables 50 and 51 show, respectively, the minimum age p and δ values in different density ranges

Table 50: Minimum age p values in different density ranges.

Density	V	r	x	y
0-0.05 ped/m^2	$4.97 \cdot 10^{-8}$	0.129	0.815	0.0613
0.05-0.1 ped/m^2	$< 10^{-8}$	0.00286	0.0232	$1.26 \cdot 10^{-6}$
0.1-0.15 ped/m^2	$8.51 \cdot 10^{-8}$	0.0207	0.00346	$7.22 \cdot 10^{-6}$
0.15-0.2 ped/m^2	0.00993	0.184	0.359	0.299
0.2-0.25 ped/m^2	0.651	0.504	0.118	0.328

Table 51: Minimum age δ values in different density ranges.

Density	V	r	x	y
0-0.05 ped/m^2	1.59	1.03	0.419	1.16
0.05-0.1 ped/m^2	1.6	0.941	0.605	1.36
0.1-0.15 ped/m^2	2.25	0.689	0.93	0.924
0.15-0.2 ped/m^2	2.22	0.987	0.652	1.1
0.2-0.25 ped/m^2	0.513	2.14	1.15	1.14

Relation

Tables 52, 53, 54 and 55 show the age dependence of observables in, respectively, colleagues, couples, families and friends. Observable values almost have no age dependence in the 20-59 years age (with the exclusion of friend velocity). It is interesting to note that the y distribution assumes a larger value for families even when only adults are involved. Another interesting, although expectable, result is that while dyads with teenagers are very abreast in the friends category, they are not abreast in the family one (the y values is almost doubled in families).

Table 52: Observable dependence on minimum age for colleagues. Lengths in millimetres, times in seconds.

Minimum age	N_g^k	V	r	x	y
20-29 years	86	1255 ± 18 (σ=165)	813 ± 20 (σ=185)	706 ± 16 (σ=144)	291 ± 24 (σ=219)
30-39 years	145	1293 ± 14 (σ=167)	861 ± 21 (σ=257)	734 ± 14 (σ=165)	331 ± 25 (σ=301)
40-49 years	71	1258 ± 14 (σ=119)	870 ± 28 (σ=236)	714 ± 19 (σ=161)	363 ± 38 (σ=324)
50-59 years	52	1274 ± 22 (σ=159)	844 ± 24 (σ=172)	700 ± 20 (σ=142)	350 ± 36 (σ=263)
60-69 years	4	1217 ± 36 (σ=72)	1075 ± 220 (σ=433)	692 ± 100 (σ=208)	617 ± 320 (σ=632)
$F_{4,353}$		1.17	1.72	0.702	1.62
p		0.326	0.146	0.591	0.168
R^2		0.013	0.0191	0.00789	0.018
δ		0.461	1.32	0.252	1.33

Table 53: Observable dependence on minimum age for couples. Lengths in millimetres, times in seconds.

Minimum age	N_g^k	V	r	x	y
10-19 years	2	958 ± 180 (σ=253)	919 ± 53 (σ=74.7)	725 ± 58 (σ=81.7)	480 ± 180 (σ=257)
20-29 years	74	1115 ± 19 (σ=165)	711 ± 27 (σ=229)	600 ± 18 (σ=154)	281 ± 28 (σ=243)
30-39 years	17	1049 ± 37 (σ=151)	670 ± 35 (σ=143)	572 ± 32 (σ=130)	274 ± 30 (σ=124)
40-49 years	3	1091 ± 110 (σ=187)	897 ± 110 (σ=198)	684 ± 66 (σ=115)	501 ± 130 (σ=223)
$F_{3,92}$		1.2	1.53	0.966	1.36
p		0.315	0.211	0.412	0.261
R^2		0.0376	0.0476	0.0306	0.0424
δ		0.946	1.78	1.19	1.65

Table 54: Observable dependence on minimum age for families. Lengths in millimetres, times in seconds.

Minimum age	N_g^k	V	r	x	y
0-9 years	31	1143 ± 42 (σ=235)	995 ± 69 (σ=383)	529 ± 34 (σ=189)	701 ± 87 (σ=485)
10-19 years	36	1163 ± 38 (σ=230)	831 ± 49 (σ=296)	617 ± 30 (σ=179)	415 ± 58 (σ=347)
20-29 years	23	1109 ± 39 (σ=187)	877 ± 58 (σ=277)	581 ± 37 (σ=177)	527 ± 78 (σ=373)
30-39 years	46	1078 ± 23 (σ=159)	814 ± 33 (σ=225)	561 ± 24 (σ=163)	458 ± 49 (σ=330)
40-49 years	41	1116 ± 31 (σ=199)	801 ± 28 (σ=181)	582 ± 23 (σ=149)	431 ± 40 (σ=256)
50-59 years	28	1048 ± 32 (σ=169)	846 ± 55 (σ=289)	562 ± 34 (σ=182)	492 ± 78 (σ=410)
60-69 years	37	1030 ± 24 (σ=145)	911 ± 63 (σ=382)	642 ± 25 (σ=154)	512 ± 75 (σ=456)
≥ 70 years	4	847 ± 52 (σ=104)	926 ± 190 (σ=384)	550 ± 38 (σ=75.6)	575 ± 240 (σ=477)
$F_{7,238}$		2.83	1.52	1.46	1.74
p		0.00758	0.162	0.182	0.101
R^2		0.0767	0.0427	0.0412	0.0486
δ		1.42	0.679	0.659	0.686

Table 55: Observable dependence on minimum age for friends. Lengths in millimetres, times in seconds.

Minimum age	N_g^k	V	r	x	y
10-19 years	23	1143 ± 61 (σ=292)	681 ± 15 (σ=73.9)	621 ± 16 (σ=78.5)	217 ± 19 (σ=93.2)
20-29 years	164	1186 ± 13 (σ=164)	801 ± 16 (σ=208)	683 ± 10 (σ=128)	298 ± 21 (σ=265)
30-39 years	56	1143 ± 28 (σ=206)	817 ± 24 (σ=178)	644 ± 22 (σ=162)	366 ± 38 (σ=286)
40-49 years	19	1089 ± 47 (σ=206)	819 ± 49 (σ=213)	682 ± 21 (σ=92.3)	341 ± 68 (σ=295)
50-59 years	22	1051 ± 36 (σ=167)	759 ± 44 (σ=208)	637 ± 24 (σ=115)	308 ± 59 (σ=276)
60-69 years	26	996 ± 38 (σ=192)	808 ± 40 (σ=202)	625 ± 33 (σ=169)	383 ± 59 (σ=299)
≥ 70 years	8	906 ± 32 (σ=91.4)	716 ± 56 (σ=159)	606 ± 18 (σ=52.2)	290 ± 85 (σ=239)
$F_{6,311}$		7.17	1.82	1.98	1.29
p		$3.58 \cdot 10^{-7}$	0.0947	0.0678	0.262
R^2		0.121	0.0339	0.0368	0.0242
δ		1.73	0.904	0.608	0.731

Gender

Tables 56, 57 and 58 show the age dependence of observables in, respectively, dyads with two females, mixed dyads and two males. The results are similar to those shown in the

main text. Although based on an extremely reduced number of data, it is interesting to note the large difference in velocity between two males and two females dyads with children (mixed dyads show a value in between, probably due to the fact that they include male and female parents), and the very large y (non-abreast formation) value assumed in two females dyads (mixed dyads on the opposite are more abreast). This values are based on very few groups, but differences are nevertheless larger than standard errors, and could reflect differences in the relation that children have with fathers and mothers (at least in the observed cultural environment).

Table 56: Observable dependence on minimum age for two females dyads. Lengths in millimetres, times in seconds.

Minimum age	N_g^k	V	r	x	y
0-9 years	6	985 ± 88 (σ=215)	1252 ± 150 (σ=378)	525 ± 78 (σ=192)	993 ± 220 (σ=535)
10-19 years	20	1075 ± 58 (σ=258)	738 ± 48 (σ=216)	621 ± 23 (σ=103)	291 ± 64 (σ=288)
20-29 years	110	1169 ± 16 (σ=167)	789 ± 21 (σ=221)	684 ± 10 (σ=107)	273 ± 26 (σ=277)
30-39 years	55	1108 ± 25 (σ=188)	777 ± 23 (σ=171)	639 ± 16 (σ=118)	330 ± 33 (σ=246)
40-49 years	24	1040 ± 33 (σ=163)	827 ± 54 (σ=266)	622 ± 25 (σ=123)	404 ± 77 (σ=379)
50-59 years	17	1015 ± 35 (σ=143)	704 ± 24 (σ=97.3)	623 ± 29 (σ=120)	240 ± 32 (σ=133)
60-69 years	17	923 ± 31 (σ=130)	791 ± 52 (σ=213)	580 ± 36 (σ=149)	390 ± 85 (σ=349)
≥ 70 years	3	958 ± 14 (σ=23.6)	629 ± 31 (σ=53.5)	587 ± 40 (σ=69)	186 ± 22 (σ=37.4)
$F_{7,244}$		6.27	4.83	3.74	5.64
p		$8.87 \cdot 10^{-7}$	$4.06 \cdot 10^{-5}$	0.000721	$4.77 \cdot 10^{-6}$
R^2		0.153	0.122	0.0969	0.139
δ		1.51	1.94	1.42	1.78

Table 57: Observable dependence on minimum age for mixed dyads. Lengths in millimetres, times in seconds.

Minimum age	N_g^k	V	r	x	y
0-9 years	12	1119 ± 56 (σ=193)	888 ± 75 (σ=259)	573 ± 61 (σ=212)	547 ± 83 (σ=287)
10-19 years	16	1060 ± 45 (σ=181)	840 ± 54 (σ=214)	620 ± 36 (σ=145)	417 ± 78 (σ=313)
20-29 years	120	1123 ± 16 (σ=175)	782 ± 23 (σ=251)	619 ± 17 (σ=182)	352 ± 27 (σ=301)
30-39 years	93	1143 ± 19 (σ=180)	834 ± 30 (σ=286)	601 ± 19 (σ=179)	435 ± 40 (σ=386)
40-49 years	53	1141 ± 26 (σ=191)	802 ± 25 (σ=178)	614 ± 21 (σ=150)	400 ± 34 (σ=245)
50-59 years	34	1078 ± 29 (σ=168)	848 ± 47 (σ=277)	604 ± 32 (σ=188)	455 ± 66 (σ=387)
60-69 years	38	1042 ± 26 (σ=160)	905 ± 61 (σ=378)	642 ± 25 (σ=152)	506 ± 73 (σ=451)
≥ 70 years	5	831 ± 44 (σ=99)	868 ± 160 (σ=363)	563 ± 33 (σ=72.8)	484 ± 210 (σ=463)
$F_{7,363}$		3.64	1.11	0.387	1.32
p		0.000822	0.358	0.91	0.24
R^2		0.0656	0.0209	0.0074	0.0248
δ		1.76	0.431	0.536	0.652

Height

Tables 59 and 60 show the age dependence of observables in, respectively, the 150-160 cm and 170-180 cm minimum height ranges. These data, which respect the patterns

Table 58: Observable dependence on minimum age for two males dyads. Lengths in millimetres, times in seconds.

Minimum age	N_g^k	V	r	x	y
0-9 years	13	1237 ± 65 (σ=233)	975 ± 120 (σ=425)	491 ± 42 (σ=153)	709 ± 150 (σ=540)
10-19 years	27	1277 ± 48 (σ=252)	803 ± 58 (σ=303)	628 ± 34 (σ=175)	376 ± 65 (σ=337)
20-29 years	134	1241 ± 14 (σ=156)	806 ± 16 (σ=180)	697 ± 13 (σ=148)	296 ± 18 (σ=208)
30-39 years	144	1280 ± 16 (σ=190)	860 ± 18 (σ=222)	732 ± 14 (σ=173)	331 ± 22 (σ=259)
40-49 years	72	1257 ± 14 (σ=122)	875 ± 27 (σ=233)	715 ± 19 (σ=159)	365 ± 39 (σ=328)
50-59 years	60	1254 ± 22 (σ=168)	846 ± 25 (σ=193)	683 ± 19 (σ=144)	373 ± 38 (σ=297)
60-69 years	12	1134 ± 48 (σ=167)	930 ± 89 (σ=307)	711 ± 54 (σ=189)	462 ± 120 (σ=406)
≥ 70 years	4	902 ± 48 (σ=95.7)	802 ± 92 (σ=183)	619 ± 19 (σ=37.6)	410 ± 140 (σ=289)
$F_{7,458}$		3.77	1.82	5.09	4.07
p		0.000553	0.081	$1.39 \cdot 10^{-5}$	0.000239
R^2		0.0544	0.0271	0.0722	0.0586
δ		2.01	0.445	1.41	1.64

highlighted in the main text, present a sufficient number of groups in each category and are thus reliable. In some situation, a noisy tracking of a child may cause to have a category with very poor and not reliable representation (e.g. groups with children but with a tall minimum height) causing some irregular behaviour in the p and δ values of tables 61 and 62.

Table 59: Observable dependence on minimum age in the 150-160 cm minimum height range. Lengths in millimetres, times in seconds.

Minimum age	N_g^k	V	r	x	y
10-19 years	21	1124 ± 54 (σ=246)	783 ± 47 (σ=215)	606 ± 31 (σ=143)	352 ± 73 (σ=333)
20-29 years	75	1157 ± 21 (σ=184)	800 ± 27 (σ=232)	668 ± 14 (σ=122)	311 ± 35 (σ=307)
30-39 years	48	1109 ± 24 (σ=168)	804 ± 32 (σ=223)	624 ± 20 (σ=139)	392 ± 44 (σ=305)
40-49 years	32	1108 ± 39 (σ=218)	828 ± 37 (σ=211)	589 ± 27 (σ=151)	452 ± 58 (σ=328)
50-59 years	19	1067 ± 39 (σ=171)	757 ± 34 (σ=146)	611 ± 36 (σ=156)	334 ± 52 (σ=228)
60-69 years	33	1008 ± 28 (σ=163)	808 ± 65 (σ=375)	641 ± 20 (σ=112)	365 ± 75 (σ=429)
≥ 70 years	5	883 ± 52 (σ=117)	666 ± 48 (σ=108)	538 ± 30 (σ=67.2)	272 ± 88 (σ=197)
$F_{6,226}$		3.65	0.427	2.13	0.849
p		0.00177	0.86	0.0506	0.533
R^2		0.0883	0.0112	0.0536	0.022
δ		1.52	0.802	1.09	0.569

Table 60: Observable dependence on minimum age in the 170-180 cm minimum height range. Lengths in millimetres, times in seconds.

Minimum age	N_g^k	V	r	x	y
20-29 years	95	1209 ± 16 (σ=152)	808 ± 18 (σ=173)	688 ± 16 (σ=156)	309 ± 22 (σ=219)
30-39 years	90	1269 ± 21 (σ=203)	838 ± 24 (σ=225)	729 ± 16 (σ=155)	300 ± 26 (σ=246)
40-49 years	45	1265 ± 18 (σ=120)	820 ± 20 (σ=137)	720 ± 20 (σ=131)	298 ± 26 (σ=176)
50-59 years	30	1241 ± 33 (σ=182)	862 ± 44 (σ=238)	635 ± 27 (σ=148)	436 ± 68 (σ=371)
$F_{3,256}$		2.21	0.709	3.36	2.61
p		0.0873	0.548	0.0194	0.0517
R^2		0.0253	0.00823	0.0379	0.0297
δ		0.339	0.282	0.613	0.51

Table 61: Minimum age p values in different minimum height ranges.

Minimum height	V	r	x	y
$<$ 140 cm	0.0333	0.137	0.0326	0.0184
140-150 cm	0.0129	0.65	0.858	0.615
150-160 cm	0.00177	0.86	0.0506	0.533
160-170 cm	0.000643	0.00561	0.807	0.00456
170-180 cm	0.0873	0.548	0.0194	0.0517
$>$ 180 cm	0.98	0.292	0.56	0.0386

Table 62: Minimum age δ values in different minimum height ranges.

Minimum height	V	r	x	y
$<$ 140 cm	0.829	0.566	0.83	0.919
140-150 cm	7.42	1.27	0.946	1.69
150-160 cm	1.52	0.802	1.09	0.569
160-170 cm	1.83	1.09	0.926	0.818
170-180 cm	0.339	0.282	0.613	0.51
$>$ 180 cm	0.181	1.2	1.53	1.26

Secondary effects and height

Density

Tables 63 and 64 show the dependence of observables on minimum height in the $0 \leq \rho \leq 0.05$ and $0.15 \leq \rho \leq 0.2$ ped/m^2 ranges, respectively. The trends discussed in the main text are still present. We notice again a tendency of short people (most probably children) to walk faster at higher density. p and δ values for minimum height at different densities are shown in tables 65 and 66.

Table 63: Observable dependence on minimum height for dyads in the $0 \leq \rho \leq 0.05$ ped/m^2 range. Lengths in millimetres, times in seconds.

Minimum height	N_g^k	V	r	x	y
< 140 cm	21	1138 ± 63 (σ=288)	1034 ± 80 (σ=368)	693 ± 44 (σ=201)	616 ± 92 (σ=421)
140-150 cm	29	1067 ± 57 (σ=304)	876 ± 51 (σ=275)	671 ± 40 (σ=218)	420 ± 64 (σ=346)
150-160 cm	148	1104 ± 18 (σ=224)	837 ± 25 (σ=304)	648 ± 11 (σ=128)	395 ± 32 (σ=390)
160-170 cm	290	1162 ± 11 (σ=187)	880 ± 19 (σ=318)	688 ± 13 (σ=217)	409 ± 22 (σ=379)
170-180 cm	141	1259 ± 16 (σ=188)	878 ± 21 (σ=253)	718 ± 17 (σ=198)	364 ± 28 (σ=331)
> 180 cm	7	1242 ± 69 (σ=182)	929 ± 73 (σ=194)	793 ± 45 (σ=120)	316 ± 100 (σ=270)
$F_{5,630}$		9.97	1.72	2.32	1.8
p		$< 10^{-8}$	0.128	0.0422	0.11
R^2		0.0733	0.0135	0.0181	0.0141
δ		0.906	0.634	1.13	0.767

Table 64: Observable dependence on minimum height for dyads in the $0.15 \leq \rho \leq 0.2$ ped/m^2 range. Lengths in millimetres, times in seconds.

Minimum height	N_g^k	V	r	x	y
< 140 cm	8	1185 ± 57 (σ=162)	872 ± 150 (σ=416)	512 ± 68 (σ=193)	555 ± 190 (σ=543)
140-150 cm	6	1166 ± 130 (σ=315)	965 ± 170 (σ=409)	604 ± 73 (σ=179)	590 ± 190 (σ=457)
150-160 cm	39	1068 ± 23 (σ=146)	754 ± 28 (σ=177)	518 ± 27 (σ=169)	408 ± 52 (σ=327)
160-170 cm	72	1093 ± 20 (σ=170)	722 ± 16 (σ=136)	586 ± 14 (σ=121)	321 ± 24 (σ=203)
170-180 cm	42	1127 ± 24 (σ=158)	792 ± 26 (σ=170)	618 ± 26 (σ=171)	352 ± 44 (σ=284)
$F_{4,162}$		1.34	3.29	2.62	2.31
p		0.259	0.0127	0.0368	0.0597
R^2		0.0319	0.0751	0.0608	0.054
δ		0.787	1.44	0.606	1.18

Relation

Tables 67, 68, 69 and 70 show the dependence of observables on minimum height for colleagues, couples, families and friends, respectively. The dependence of observables on height appears to be attenuated when analysed for groups with a fixed relation (and in particular for couples), as shown by the higher p values, and, to a lesser extent, lower δ values.

Table 65: p values for minimum height in different density ranges.

Density	V	r	x	y
0-0.05 ped/m^2	$< 10^{-8}$	0.128	0.0422	0.11
0.05-0.1 ped/m^2	$< 10^{-8}$	0.000607	0.000112	$4.09 \cdot 10^{-6}$
0.1-0.15 ped/m^2	$1.84 \cdot 10^{-5}$	$< 10^{-8}$	$3.34 \cdot 10^{-5}$	$< 10^{-8}$
0.15-0.2 ped/m^2	0.259	0.0127	0.0368	0.0597
0.2-0.25 ped/m^2	0.303	0.602	0.603	0.765

Table 66: δ values for minimum height in different density ranges.

Density	V	r	x	y
0-0.05 ped/m^2	0.906	0.634	1.13	0.767
0.05-0.1 ped/m^2	1.17	0.664	0.63	0.973
0.1-0.15 ped/m^2	0.856	1.32	1.12	1.29
0.15-0.2 ped/m^2	0.787	1.44	0.606	1.18
0.2-0.25 ped/m^2	0.886	0.578	0.54	0.422

Table 67: Observable dependence on minimum height for colleague dyads. Lengths in millimetres, times in seconds.

Minimum height	N_g^k	V	r	x	y
150-160 cm	15	1135 ± 36 (σ=141)	732 ± 25 (σ=98.2)	652 ± 22 (σ=85.9)	265 ± 31 (σ=120)
160-170 cm	159	1276 ± 12 (σ=157)	874 ± 21 (σ=265)	712 ± 13 (σ=168)	369 ± 28 (σ=351)
170-180 cm	170	1288 ± 12 (σ=155)	846 ± 15 (σ=202)	731 ± 12 (σ=153)	312 ± 18 (σ=236)
> 180 cm	13	1220 ± 36 (σ=128)	789 ± 59 (σ=214)	689 ± 33 (σ=120)	263 ± 67 (σ=241)
$F_{3,353}$		5.03	2.2	1.49	1.63
p		0.002	0.0881	0.217	0.182
R^2		0.041	0.0183	0.0125	0.0137
δ		0.996	0.556	0.53	0.307

Table 68: Observable dependence on minimum height for couples. Lengths in millimetres, times in seconds.

Minimum height	N_g^k	V	r	x	y
150-160 cm	20	1060 ± 43 (σ=193)	736 ± 32 (σ=143)	631 ± 25 (σ=111)	288 ± 39 (σ=175)
160-170 cm	60	1114 ± 21 (σ=160)	716 ± 33 (σ=254)	591 ± 22 (σ=171)	305 ± 34 (σ=261)
170-180 cm	15	1092 ± 42 (σ=162)	678 ± 35 (σ=137)	592 ± 24 (σ=93.1)	245 ± 39 (σ=151)
$F_{2,92}$		0.773	0.296	0.528	0.408
p		0.464	0.745	0.591	0.666
R^2		0.0165	0.00639	0.0114	0.00878
δ		0.321	0.414	0.249	0.249

Gender

Tables 71, 72 and 73 show the dependence of observables on minimum height for two females, mixed and two males dyads, respectively. As discussed in the main text and

Table 69: Observable dependence on minimum height for families. Lengths in millimetres, times in seconds.

Minimum height	N_g^k	V	r	x	y
< 140 cm	33	1117 ± 38 (σ=216)	1062 ± 72 (σ=411)	570 ± 39 (σ=222)	746 ± 89 (σ=509)
140-150 cm	19	1122 ± 62 (σ=270)	832 ± 60 (σ=262)	636 ± 32 (σ=139)	410 ± 65 (σ=285)
150-160 cm	77	1107 ± 23 (σ=204)	835 ± 34 (σ=302)	583 ± 19 (σ=163)	466 ± 44 (σ=387)
160-170 cm	99	1080 ± 17 (σ=170)	831 ± 24 (σ=241)	580 ± 17 (σ=166)	467 ± 33 (σ=332)
170-180 cm	17	1053 ± 36 (σ=149)	833 ± 65 (σ=268)	566 ± 37 (σ=153)	458 ± 98 (σ=403)
$F_{4,240}$		0.602	4.3	0.542	4.04
p		0.661	0.00224	0.705	0.00344
R^2		0.00993	0.0668	0.00896	0.0631
δ		0.312	0.788	0.478	0.76

Table 70: Observable dependence on minimum height for friends. Lengths in millimetres, times in seconds.

Minimum height	N_g^k	V	r	x	y
< 140 cm	4	1129 ± 55 (σ=109)	611 ± 20 (σ=39.7)	518 ± 31 (σ=61.8)	246 ± 68 (σ=136)
140-150 cm	16	1115 ± 88 (σ=354)	816 ± 44 (σ=175)	628 ± 43 (σ=172)	382 ± 80 (σ=319)
150-160 cm	101	1100 ± 20 (σ=202)	770 ± 21 (σ=209)	659 ± 10 (σ=104)	287 ± 27 (σ=269)
160-170 cm	142	1138 ± 15 (σ=174)	802 ± 18 (σ=211)	665 ± 12 (σ=146)	324 ± 23 (σ=276)
170-180 cm	53	1199 ± 23 (σ=168)	806 ± 19 (σ=138)	673 ± 18 (σ=132)	323 ± 32 (σ=233)
> 180 cm	2	1606 ± 23 (σ=32.1)	928 ± 61 (σ=86.6)	805 ± 120 (σ=171)	329 ± 72 (σ=102)
$F_{5,312}$		4.13	1.27	1.68	0.515
p		0.0012	0.276	0.138	0.765
R^2		0.0621	0.02	0.0263	0.00819
δ		2.52	5.74	2.83	0.461

shown in the supplementary figure S8, there is a loss of linearity in x, but the patterns described in the main text are still present, although partially attenuated, when gender is kept fixed.

Table 71: Observable dependence on minimum height for 2 female dyads. Lengths in millimetres, times in seconds.

Minimum height	N_g^k	V	r	x	y
< 140 cm	7	956 ± 74 (σ=195)	1186 ± 150 (σ=385)	529 ± 68 (σ=180)	935 ± 190 (σ=509)
140-150 cm	21	1022 ± 57 (σ=262)	841 ± 66 (σ=300)	591 ± 36 (σ=166)	439 ± 96 (σ=442)
150-160 cm	114	1098 ± 18 (σ=197)	780 ± 20 (σ=214)	656 ± 11 (σ=112)	306 ± 26 (σ=279)
160-170 cm	104	1131 ± 16 (σ=159)	768 ± 18 (σ=185)	658 ± 11 (σ=115)	278 ± 24 (σ=245)
170-180 cm	6	1123 ± 77 (σ=188)	706 ± 34 (σ=83.3)	644 ± 26 (σ=64.8)	213 ± 59 (σ=144)
$F_{4,247}$		2.58	6.59	3.11	9.37
p		0.0381	$4.66 \cdot 10^{-5}$	0.0159	$4.55 \cdot 10^{-7}$
R^2		0.0401	0.0965	0.048	0.132
δ		1.08	1.66	1.08	1.86

Table 72: Observable dependence on minimum height for mixed gender dyads. Lengths in millimetres, times in seconds.

Minimum height	N_g^k	V	r	x	y
< 140 cm	14	1100 ± 42 (σ=159)	947 ± 82 (σ=307)	590 ± 53 (σ=199)	593 ± 100 (σ=373)
140-150 cm	9	1107 ± 66 (σ=199)	967 ± 91 (σ=273)	664 ± 42 (σ=127)	552 ± 120 (σ=346)
150-160 cm	99	1092 ± 20 (σ=195)	829 ± 29 (σ=286)	609 ± 16 (σ=160)	429 ± 38 (σ=376)
160-170 cm	210	1128 ± 12 (σ=176)	811 ± 18 (σ=262)	618 ± 13 (σ=184)	395 ± 23 (σ=328)
170-180 cm	37	1083 ± 28 (σ=172)	806 ± 44 (σ=267)	589 ± 24 (σ=143)	404 ± 61 (σ=372)
> 180 cm	2	937 ± 140 (σ=204)	687 ± 3.2 (σ=4.56)	573 ± 48 (σ=67.8)	253 ± 55 (σ=77.2)
$F_{5,365}$		1.14	1.3	0.418	1.27
p		0.34	0.262	0.836	0.277
R^2		0.0153	0.0175	0.00569	0.0171
δ		1.08	1.09	0.751	0.945

Table 73: Observable dependence on minimum height for male dyads. Lengths in millimetres, times in seconds.

Minimum height	N_g^k	V	r	x	y
< 140 cm	18	1221 ± 48 (σ=203)	977 ± 110 (σ=455)	577 ± 53 (σ=226)	631 ± 130 (σ=549)
140-150 cm	9	1301 ± 140 (σ=405)	861 ± 85 (σ=255)	640 ± 47 (σ=142)	456 ± 110 (σ=343)
150-160 cm	21	1196 ± 39 (σ=180)	739 ± 36 (σ=164)	600 ± 22 (σ=103)	320 ± 57 (σ=261)
160-170 cm	184	1238 ± 13 (σ=179)	863 ± 18 (σ=241)	700 ± 13 (σ=174)	372 ± 23 (σ=315)
170-180 cm	219	1272 ± 11 (σ=156)	834 ± 12 (σ=184)	719 ± 10 (σ=151)	310 ± 15 (σ=224)
> 180 cm	15	1271 ± 46 (σ=178)	808 ± 53 (σ=207)	705 ± 35 (σ=134)	272 ± 59 (σ=229)
$F_{5,460}$		1.46	2.56	4.51	5.03
p		0.202	0.0267	0.000499	0.00017
R^2		0.0156	0.0271	0.0468	0.0518
δ		0.394	0.721	0.903	0.826

Age

Tables 74 and 75 show the dependence on minimum height of all observables for dyads with minimum age in the 20-29 and 50-59 year ranges, respectively, showing the effect of removing children from the population. Finally, tables 76 and 77 show the dependence of, respectively, minimum height p and δ values on minimum age ranges.

Table 74: Observable dependence on minimum height for dyads with minimum age in the 20-29 year range. Lengths in millimetres, times in seconds.

Minimum height	N_g^k	V	r	x	y
140-150 cm	2	1161 ± 180 (σ=254)	748 ± 62 (σ=87.2)	613 ± 41 (σ=58.5)	398 ± 54 (σ=75.7)
150-160 cm	75	1157 ± 21 (σ=184)	800 ± 27 (σ=232)	668 ± 14 (σ=122)	311 ± 35 (σ=307)
160-170 cm	188	1175 ± 13 (σ=176)	781 ± 17 (σ=231)	658 ± 12 (σ=165)	300 ± 19 (σ=265)
170-180 cm	95	1209 ± 16 (σ=152)	808 ± 18 (σ=173)	688 ± 16 (σ=156)	309 ± 22 (σ=219)
> 180 cm	3	1262 ± 130 (σ=223)	974 ± 170 (σ=300)	625 ± 9.5 (σ=16.4)	556 ± 220 (σ=373)
$F_{4,358}$		1.19	0.818	0.684	0.756
p		0.315	0.514	0.603	0.554
R^2		0.0131	0.00906	0.00759	0.00838
δ		0.567	0.906	0.482	0.962

Table 75: Observable dependence on minimum height for dyads with average age in the 50-59 year range. Lengths in millimetres, times in seconds.

Minimum height	N_g^k	V	r	x	y
140-150 cm	3	1043 ± 41 (σ=70.2)	784 ± 56 (σ=97)	746 ± 63 (σ=109)	190 ± 16 (σ=28)
150-160 cm	19	1067 ± 39 (σ=171)	757 ± 34 (σ=146)	611 ± 36 (σ=156)	334 ± 52 (σ=228)
160-170 cm	59	1162 ± 25 (σ=191)	830 ± 29 (σ=226)	664 ± 22 (σ=165)	371 ± 41 (σ=315)
170-180 cm	30	1241 ± 33 (σ=182)	862 ± 44 (σ=238)	635 ± 27 (σ=148)	436 ± 68 (σ=371)
$F_{3,107}$		3.84	0.928	0.974	0.806
p		0.0118	0.43	0.408	0.493
R^2		0.0972	0.0254	0.0266	0.0221
δ		1.12	0.502	0.886	0.687

Table 76: Minimum height p values in different minimum age ranges.

Minimum age	V	r	x	y
0-9 years	0.000332	0.143	0.662	0.127
10-19 years	0.311	0.822	0.478	0.926
20-29 years	0.315	0.514	0.603	0.554
30-39 years	$5 \cdot 10^{-7}$	0.595	0.00388	0.0423
40-49 years	0.000142	0.489	0.00545	0.0649
50-59 years	0.0118	0.43	0.408	0.493
60-69 years	0.091	0.23	0.24	0.182
$\geq$ 70 years	0.627	0.0424	0.0506	0.107

Table 77: Minimum height δ values in different minimum age ranges.

Minimum age	V	r	x	y
0-9 years	3.42	1.11	2	1.04
10-19 years	0.883	0.346	0.519	0.275
20-29 years	0.567	0.906	0.482	0.962
30-39 years	2.32	0.943	0.702	1.28
40-49 years	2.48	0.671	0.993	0.944
50-59 years	1.12	0.502	0.886	0.687
60-69 years	0.89	0.436	0.645	0.597
$\geq$ 70 years	1.08	2.05	2.01	1.7
