# Supplementary material for: Intrinsic group behaviour: Dependence of pedestrian dyad dynamics on principal social and personal features
Source: PLoS One. 2017 Nov 2;12(11):e0187253. doi: 10.1371/journal.pone.0187253 (PMC5667819; doi:10.1371/journal.pone.0187253)
Supplement: S4 Appendix — (PDF) [file pone.0187253.s013.pdf]

Coder reliability

Analysis of coder agreement

We consider a few possible statistical indicators of agreement between coders.

Cohen's κ

Cohen's κ [1] is a very popular indicator to compare the agreement between two coders, based on the equation

$$\kappa = \frac{p - p_r}{1 - p_r}, \tag{1}$$

where p stands for the agreement rate between coders and p_r for the probability of random agreement. The agreement between pairs of coders according to Cohen's κ is shown in table 1.

Table 1: Agreement between pairs of coders according to Cohen's κ statistics. $C_i - C_j$ stands for agreement between coder i and j.

Pair	Purpose	Gender	Relation	Min Age	Avg Age	Max Age
$C_1 - C_2$	0.815	0.961	0.636	0.476	0.582	0.555
$C_1 - C_3$	0.923	0.978	0.728	0.808	0.839	0.866
$C_2 - C_3$	0.810	0.944	0.647	0.449	0.508	0.526

These results show that in general the agreement is higher for gender, followed by purpose and relation. The agreement between coders 1 and 3 is similar also concerning age, while the agreement with coder 2 is quite poor in these categories. Although there is no real sound mathematical way to evaluate the absolute value of these numbers, according to popular benchmarks, an agreement between 0.8 and 1 is considered as "almost perfect", an agreement between 0.6 and 0.8 as "substantial", while an agreement between 0.4 and 0.6 is only "moderate"[2].

Fleiss' κ

It generalises eq. 1 to deal with multiple coders and categories[3]. The corresponding values are shown in table 2.

We see that, in relative terms, agreement is higher for gender, followed by purpose and relation, and lowest for age. In absolute terms, according to the benchmarks, we have almost perfect agreement in gender and purpose, substantial in relation and "fair"

[1] Cohen, Jacob *A coefficient of agreement for nominal scales.* Educational and Psychological Measurement 20 (1): 3746 (1960), citation [50] in the main text.

[2] Landis, J.R.; Koch, G.G. (1977). *The measurement of observer agreement for categorical data* Biometrics. 33 (1): 159174, citation [51] in the main text.

[3] Fleiss, J. L. (1971) *Measuring nominal scale agreement among many raters*, Psychological Bulletin, Vol. 76, No. 5 pp. 378–382, citation [52] in the main text.

Table 2: Agreement between coders according to Fleiss' κ statistics.

Purpose	Gender	Relation	Min Age	Avg Age	Max Age
0.849	0.961	0.669	0.289	0.332	0.300

(i.e., worst than "moderate") for age indicators, due to the effect of the different coding by coder 2.

Anyway, if we try to plot the age difference between coders, as in figure 1, we see that although disagreement with coder 2 is substantial, it is almost completely limited to a tendency of coder 2 to put pedestrians in a slightly younger category, i.e. the difference in age between the codings is limited. Nevertheless the Fleiss indicator does not take in account the magnitude of difference, and is thus not completely adequate to deal with ordered data.

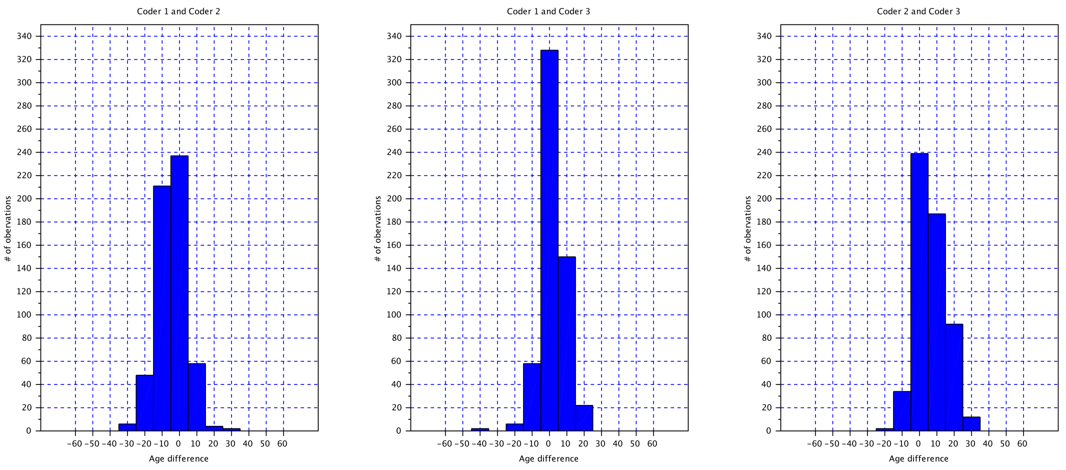

Figure 1: Histograms of age differences between coders.

Krippendorff's α

The Krippendorff α statistics[4], that allows for consideration of quantitative differences between coding results, gives the results shown in table 3.

Krippendorff does not provide any "magic number" but suggests to use data with at least $\alpha > 0.667$ (satisfied by all our categories) and require $\alpha > 0.8$, satisfied by purpose and gender, for reliable results (α between 0.667 and 0.8 could be used for "tentative

[4]Krippendorff, Klaus. *Reliability in content analysis*, Human communication research 30.3 (2004): 411-433, citation [53] in the main text.

Table 3: Agreement between coders according to Krippendorff's α statistics. Purpose, gender and relation are "nominal" data, age is on an "interval", according to the definition of α statistics.

Purpose	Gender	Relation	Min Age	Avg Age	Max Age
0.849	0.961	0.669	0.709	0.730	0.729

conclusions").

Discussion

Using popular indicators of coder reliability, we have found that, in relative terms, the most reliable coding regards gender, followed by purpose. In absolute terms, according to the Krippendorff α statistics that can better cope with the nature of our data, we may see that the purpose and gender codings may be considered as enough reliable to provide sound findings, while the relation and age codings are reliable enough for reporting tentative findings.

The analysis based on these indicators provides an estimate on the reliability of coding of pedestrians in different categories. We may nevertheless use another approach to test the reliability of our findings when based on different coding processes. Since for each category we analyse the values of the observables V, r, x and y, we may compare these quantitative results between different coders.

This comparison, which has also the advantage of being based on more mathematically sound statistical indicators (standard errors, ANOVA analysis) is performed in the following section, and shows again that for purpose and gender we have an almost perfect quantitative agreement, while for relation and age, although the agreement is less good, the major patterns of behaviour are qualitatively observed regardless of coders.

Quantitative comparison of results

Purpose

The results (on the common subset of data) for the purpose dependence of all observables between the main coder (coder 1) and the secondary coders are compared in tables 4, 5 and 6.

The differences between coders are thus always of one standard error or smaller, and the extremely significant statistical differences in the x and V distribution (along with the less significant y and r ones) are reported by all coders.

Relation

The results (on the common subset of data) for the relation dependence of all observables between the main coder (coder 1) and the secondary coders are compared in tables 7,

Table 4: Observable dependence on purpose for dyads according to coder 1 (common data set only). Lengths in millimetres, times in seconds.

Purpose	N_g^k	V	r	x	y
Leisure	136	1085 ± 19 (σ=220)	796 ± 21 (σ=248)	636 ± 13 (σ=151)	351 ± 28 (σ=327)
Work	132	1257 ± 14 (σ=157)	829 ± 17 (σ=196)	723 ± 12 (σ=143)	303 ± 21 (σ=241)
$F_{1,266}$		53.1	1.41	23.5	1.88
p		$< 10^{-8}$	0.236	$2.14{\cdot}10^{-6}$	0.171
R^2		0.166	0.00529	0.0811	0.00703
δ		0.893	0.146	0.594	0.168

Table 5: Observable dependence on purpose for dyads according to coder 2 (common data set only). Lengths in millimetres, times in seconds.

Purpose	N_g^k	V	r	x	y
Leisure	151	1093 ± 17 (σ=212)	793 ± 20 (σ=243)	641 ± 12 (σ=147)	344 ± 26 (σ=318)
Work	117	1269 ± 15 (σ=159)	837 ± 18 (σ=196)	728 ± 13 (σ=146)	306 ± 22 (σ=243)
$F_{1,266}$		56.2	2.56	23.4	1.13
p		$< 10^{-8}$	0.111	$2.18{\cdot}10^{-6}$	0.289
R^2		0.175	0.00954	0.081	0.00422
δ		0.927	0.198	0.599	0.131

Table 6: Observable dependence on purpose for dyads according to coder 3 (common data set only). Lengths in millimetres, times in seconds.

Purpose	N_g^k	V	r	x	y
Leisure	133	1077 ± 19 (σ=217)	789 ± 22 (σ=250)	626 ± 13 (σ=145)	354 ± 29 (σ=330)
Work	133	1262 ± 14 (σ=156)	836 ± 17 (σ=195)	732 ± 12 (σ=144)	302 ± 21 (σ=239)
$F_{1,264}$		63.6	2.93	35.6	2.13
p		$< 10^{-8}$	0.0881	$< 10^{-8}$	0.145
R^2		0.194	0.011	0.119	0.00802
δ		0.982	0.211	0.734	0.18

8 and 9. While all the major trends exposed in the main text are confirmed, quantitative results between coders may sometimes be different (we refer in particular to the y distribution for couples, extremely narrow according to coder 3).

Gender

The results (on the common subset of data) for the gender dependence of all observables between the main coder (coder 1) and the secondary coders are compared in tables 10, 11 and 12, showing that there is basically no difference in the coding of gender.

Table 7: Observable dependence on relation for dyads according to coder 1 (common data set only). Lengths in millimetres, times in seconds.

Relation	N_g^k	V	r	x	y
Colleagues	125	1256 ± 14 (σ=154)	829 ± 18 (σ=196)	725 ± 13 (σ=142)	301 ± 21 (σ=239)
Couples	28	1087 ± 37 (σ=194)	690 ± 33 (σ=174)	611 ± 21 (σ=112)	248 ± 37 (σ=198)
Families	40	1051 ± 24 (σ=153)	864 ± 54 (σ=341)	594 ± 21 (σ=134)	492 ± 69 (σ=438)
Friends	56	1121 ± 36 (σ=271)	777 ± 24 (σ=182)	669 ± 19 (σ=145)	286 ± 32 (σ=243)
$F_{3,245}$		16.4	4.19	11.8	6.12
p		$< 10^{-8}$	0.00651	$3.06 \cdot 10^{-7}$	0.0005
R^2		0.167	0.0488	0.126	0.0697
δ		1.33	0.612	0.934	0.678

Table 8: Observable dependence on relation for dyads according to coder 2 (common data set only). Lengths in millimetres, times in seconds.

Relation	N_g^k	V	r	x	y
Colleagues	116	1267 ± 14 (σ=156)	839 ± 18 (σ=197)	729 ± 14 (σ=147)	308 ± 23 (σ=244)
Couples	44	1082 ± 28 (σ=184)	703 ± 21 (σ=140)	582 ± 19 (σ=125)	296 ± 33 (σ=221)
Families	42	1054 ± 25 (σ=164)	894 ± 53 (σ=341)	651 ± 25 (σ=163)	451 ± 70 (σ=457)
Friends	66	1131 ± 31 (σ=254)	786 ± 23 (σ=188)	673 ± 17 (σ=136)	304 ± 29 (σ=238)
$F_{3,264}$		19	6.55	11.9	3.13
p		$< 10^{-8}$	0.000276	$2.54 \cdot 10^{-7}$	0.0262
R^2		0.178	0.0692	0.119	0.0344
δ		1.35	0.74	1.04	0.437

Table 9: Observable dependence on relation for dyads according to coder 3 (common data set only) Lengths in millimetres, times in seconds..

Relation	N_g^k	V	r	x	y
Colleagues	136	1259 ± 14 (σ=158)	834 ± 17 (σ=194)	727 ± 13 (σ=147)	304 ± 21 (σ=242)
Couples	23	1070 ± 42 (σ=204)	624 ± 20 (σ=96.4)	578 ± 20 (σ=95.1)	182 ± 17 (σ=81.2)
Families	50	1053 ± 24 (σ=172)	867 ± 44 (σ=312)	612 ± 20 (σ=140)	478 ± 59 (σ=416)
Friends	54	1084 ± 32 (σ=235)	780 ± 27 (σ=196)	663 ± 22 (σ=159)	298 ± 33 (σ=245)
$F_{3,259}$		23.4	7.57	12.4	7.52
p		$< 10^{-8}$	$7.11 \cdot 10^{-5}$	$1.36 \cdot 10^{-7}$	$7.61 \cdot 10^{-5}$
R^2		0.213	0.0807	0.125	0.0801
δ		1.27	0.915	1.06	0.849

Age

The results (on the common subset of data) for the minimum age dependence of all observables between the main coder (coder 1) and the secondary coders are compared in tables 13, 14 and 15. Sadly, almost no groups with children are present in the common set. The drop in velocity with age is, on the other hand, confirmed in a statistically significant way by all coders.

Table 10: Observable dependence on gender for dyads according to coder 1 (common data set only). Lengths in millimetres, times in seconds.

Gender	N_g^k	V	r	x	y
Two females	55	1076 ± 32 (σ=240)	745 ± 21 (σ=155)	629 ± 15 (σ=112)	290 ± 33 (σ=242)
Mixed	86	1095 ± 19 (σ=173)	820 ± 31 (σ=287)	641 ± 17 (σ=159)	384 ± 39 (σ=360)
Two males	127	1261 ± 16 (σ=178)	836 ± 17 (σ=195)	727 ± 13 (σ=150)	305 ± 22 (σ=243)
$F_{2,265}$		27.2	3.25	12.8	2.48
p		$< 10^{-8}$	0.0404	$5.09 \cdot 10^{-6}$	0.0855
R^2		0.171	0.0239	0.0879	0.0184
δ		0.93	0.494	0.699	0.292

Table 11: Observable dependence on gender for dyads according to coder 2 (common data set only). Lengths in millimetres, times in seconds.

Gender	N_g^k	V	r	x	y
Two females	53	1078 ± 33 (σ=241)	747 ± 22 (σ=158)	637 ± 15 (σ=106)	286 ± 32 (σ=233)
Mixed	89	1093 ± 18 (σ=173)	814 ± 30 (σ=283)	635 ± 17 (σ=159)	382 ± 38 (σ=360)
Two males	126	1263 ± 16 (σ=177)	838 ± 17 (σ=194)	728 ± 13 (σ=150)	306 ± 22 (σ=244)
$F_{2,265}$		28.2	3.12	13.3	2.48
p		$< 10^{-8}$	0.0459	$3.22 \cdot 10^{-6}$	0.0853
R^2		0.176	0.023	0.091	0.0184
δ		0.935	0.494	0.604	0.3

Table 12: Observable dependence on gender for dyads according to coder 3 (common data set only). Lengths in millimetres, times in seconds.

Gender	N_g^k	V	r	x	y
Two females	55	1074 ± 32 (σ=239)	742 ± 21 (σ=153)	636 ± 14 (σ=103)	281 ± 31 (σ=230)
Mixed	89	1093 ± 19 (σ=175)	824 ± 31 (σ=288)	634 ± 17 (σ=161)	397 ± 39 (σ=368)
Two males	124	1267 ± 16 (σ=173)	834 ± 17 (σ=190)	730 ± 13 (σ=150)	298 ± 21 (σ=232)
$F_{2,265}$		30.4	3.44	14.2	3.99
p		$< 10^{-8}$	0.0336	$1.36 \cdot 10^{-6}$	0.0196
R^2		0.187	0.0253	0.0969	0.0293
δ		0.987	0.511	0.622	0.359

Table 13: Observable dependence on minimum age for dyads according to coder 1 (common data set only). Lengths in millimetres, times in seconds.

Minimum age	N_g^k	V	r	x	y
10-19 years	16	1157 ± 86 (σ=343)	715 ± 31 (σ=123)	653 ± 23 (σ=92.3)	223 ± 38 (σ=151)
20-29 years	58	1183 ± 28 (σ=215)	765 ± 28 (σ=211)	666 ± 20 (σ=149)	268 ± 33 (σ=252)
30-39 years	96	1186 ± 21 (σ=203)	817 ± 21 (σ=211)	689 ± 17 (σ=166)	327 ± 27 (σ=262)
40-49 years	41	1193 ± 25 (σ=161)	811 ± 27 (σ=173)	684 ± 22 (σ=143)	327 ± 38 (σ=245)
50-59 years	31	1210 ± 29 (σ=160)	880 ± 46 (σ=254)	696 ± 29 (σ=160)	407 ± 65 (σ=360)
60-69 years	21	1017 ± 35 (σ=160)	869 ± 66 (σ=304)	671 ± 34 (σ=156)	401 ± 85 (σ=388)
≥ 70 years	5	949 ± 15 (σ=34.2)	913 ± 170 (σ=379)	608 ± 28 (σ=61.7)	551 ± 210 (σ=470)
$F_{6,261}$		3.35	1.81	0.462	1.91
p		0.00337	0.0974	0.836	0.0789
R^2		0.0715	0.0399	0.0105	0.0421
δ		1.73	0.964	0.578	1.29

Table 14: Observable dependence on minimum age for dyads according to coder 2 (common data set only). Lengths in millimetres, times in seconds.

Minimum age	N_g^k	V	r	x	y
0-9 years	2	1190 ± 220 (σ=312)	749 ± 110 (σ=152)	700 ± 87 (σ=123)	202 ± 55 (σ=77.8)
10-19 years	16	1169 ± 84 (σ=334)	682 ± 20 (σ=80.1)	646 ± 20 (σ=78.4)	172 ± 18 (σ=73.3)
20-29 years	107	1163 ± 19 (σ=196)	765 ± 16 (σ=165)	655 ± 13 (σ=138)	288 ± 22 (σ=231)
30-39 years	78	1217 ± 24 (σ=209)	869 ± 28 (σ=244)	727 ± 21 (σ=185)	362 ± 33 (σ=290)
40-49 years	32	1181 ± 31 (σ=176)	855 ± 44 (σ=249)	645 ± 22 (σ=124)	418 ± 66 (σ=373)
50-59 years	24	1074 ± 32 (σ=158)	853 ± 60 (σ=293)	706 ± 29 (σ=143)	343 ± 74 (σ=363)
60-69 years	9	1047 ± 42 (σ=127)	861 ± 100 (σ=311)	655 ± 45 (σ=135)	432 ± 120 (σ=375)
$F_{6,261}$		2.1	3.09	2.3	2.13
p		0.0533	0.0061	0.0349	0.0505
R^2		0.0461	0.0663	0.0503	0.0467
δ		0.842	0.833	0.482	1.14

Table 15: Observable dependence on minimum age for dyads according to coder 3 (common data set only). Lengths in millimetres, times in seconds.

Minimum age	N_g^k	V	r	x	y
10-19 years	14	1163 ± 98 (σ=367)	701 ± 31 (σ=117)	623 ± 35 (σ=130)	218 ± 48 (σ=181)
20-29 years	64	1157 ± 29 (σ=236)	758 ± 24 (σ=194)	658 ± 20 (σ=158)	274 ± 27 (σ=220)
30-39 years	50	1197 ± 27 (σ=193)	830 ± 32 (σ=227)	685 ± 23 (σ=162)	349 ± 43 (σ=302)
40-49 years	77	1205 ± 19 (σ=163)	832 ± 23 (σ=205)	684 ± 16 (σ=141)	351 ± 33 (σ=293)
50-59 years	36	1205 ± 25 (σ=152)	820 ± 35 (σ=207)	722 ± 28 (σ=168)	300 ± 39 (σ=233)
60-69 years	20	1025 ± 40 (σ=179)	903 ± 74 (σ=332)	699 ± 29 (σ=129)	418 ± 97 (σ=436)
≥ 70 years	7	956 ± 26 (σ=69.1)	881 ± 120 (σ=326)	605 ± 36 (σ=96.4)	503 ± 140 (σ=382)
$F_{6,261}$		3.69	2.04	1.33	1.67
p		0.00153	0.0604	0.245	0.129
R^2		0.0783	0.0449	0.0296	0.0369
δ		1.74	0.755	0.732	1.09
