# Supplementary material for: Intrinsic group behaviour: Dependence of pedestrian dyad dynamics on principal social and personal features
Source: PLoS One. 2017 Nov 2;12(11):e0187253. doi: 10.1371/journal.pone.0187253 (PMC5667819; doi:10.1371/journal.pone.0187253)
Supplement: S5 Appendix — (PDF) [file pone.0187253.s014.pdf]

Dependence on average and maximum age

Average age

Table 1 shows the average age dependence of all observables, and the age dependence of variables r, x, y is also graphically shown in figure 1, while that of V is shown in figure 2 (left panels).

Table 1: Observable dependence on average age for dyads. Lengths in millimetres, times in seconds.

Average age	N_g^k	V	r	x	y
10-19 years	60	1147 ± 34 (σ=264)	865 ± 43 (σ=332)	575 ± 20 (σ=158)	496 ± 57 (σ=445)
20-29 years	370	1181 ± 9.2 (σ=178)	793 ± 12 (σ=226)	662 ± 8.1 (σ=155)	313 ± 14 (σ=274)
30-39 years	269	1213 ± 12 (σ=199)	831 ± 14 (σ=234)	670 ± 11 (σ=174)	360 ± 18 (σ=302)
40-49 years	195	1172 ± 13 (σ=183)	852 ± 17 (σ=232)	674 ± 12 (σ=167)	387 ± 23 (σ=316)
50-59 years	114	1157 ± 18 (σ=194)	825 ± 20 (σ=217)	650 ± 15 (σ=159)	376 ± 30 (σ=317)
60-69 years	69	1032 ± 20 (σ=168)	875 ± 40 (σ=332)	635 ± 20 (σ=163)	467 ± 50 (σ=416)
≥ 70 years	12	886 ± 29 (σ=99.8)	786 ± 79 (σ=275)	588 ± 19 (σ=66.6)	385 ± 100 (σ=363)
$F_{6,1082}$		13.2	2.26	3.79	4.75
p		$< 10^{-8}$	0.036	0.000955	$8.72 \cdot 10^{-5}$
R^2		0.0681	0.0124	0.0206	0.0257
δ		1.67	0.275	0.598	0.603

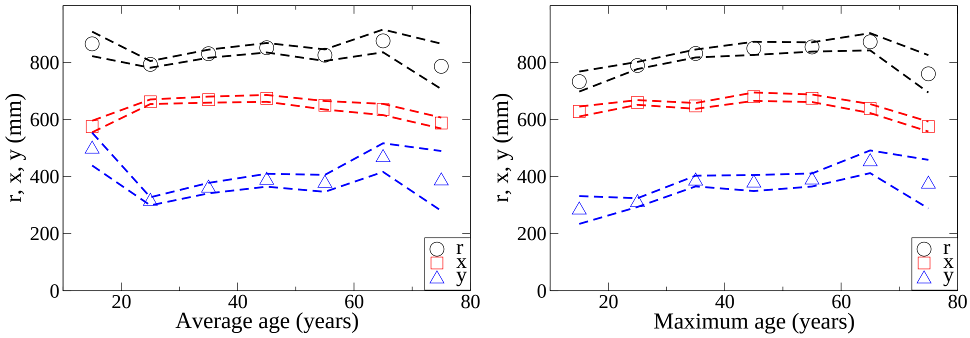

Figure 1: r (black and circles), x (red and squares) and y (blue and triangles) dependence on average (left) and maximum (right) age. Dashed lines provide standard error confidence bars. The point at 75 years corresponds to the "70 years or more" slot.

Maximum age

Table 2 shows the average age dependence of all observables, and the age dependence of variables r, x, y is also graphically shown in figure 1, while that of V is shown in figure

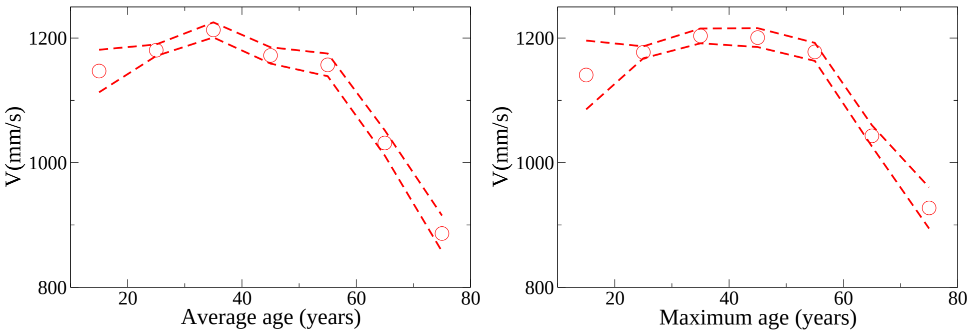

Figure 2: V dependence on average (left) and maximum (right) age. Dashed lines provide standard error confidence bars. The point at 75 years corresponds to the "70 years or more" slot.

2 (right panels).

Table 2: Observable dependence on maximum age for dyads. Lengths in millimetres, times in seconds.

Maximum age	N_g^k	V	r	x	y
10-19 years	28	1141 ± 55 (σ=292)	733 ± 35 (σ=186)	628 ± 17 (σ=92.5)	283 ± 49 (σ=258)
20-29 years	327	1177 ± 9.6 (σ=174)	789 ± 12 (σ=225)	660 ± 8.2 (σ=149)	309 ± 15 (σ=278)
30-39 years	292	1203 ± 12 (σ=204)	831 ± 14 (σ=238)	648 ± 10 (σ=172)	384 ± 19 (σ=321)
40-49 years	143	1201 ± 15 (σ=181)	849 ± 23 (σ=275)	680 ± 15 (σ=176)	377 ± 28 (σ=336)
50-59 years	179	1178 ± 14 (σ=193)	854 ± 16 (σ=217)	674 ± 13 (σ=178)	388 ± 23 (σ=306)
60-69 years	105	1043 ± 17 (σ=174)	872 ± 30 (σ=310)	638 ± 16 (σ=162)	452 ± 40 (σ=407)
$\geq$ 70 years	15	927 ± 33 (σ=128)	760 ± 65 (σ=254)	575 ± 18 (σ=67.9)	374 ± 85 (σ=330)
$F_{6,1082}$		14.2	3.37	1.97	3.7
p		$< 10^{-8}$	0.0027	0.0668	0.00122
R^2		0.0731	0.0183	0.0108	0.0201
δ		1.38	0.484	0.619	0.443

Discussion

It may be seen that the results concerning maximum and average and minimum (shown in the main text) age are quite similar above 20 years. Nevertheless, using minimum age allows us to spot the presence of children below 10 years of age and verify their peculiar behaviour.
