# Supplementary material for: Intrinsic group behaviour: Dependence of pedestrian dyad dynamics on principal social and personal features
Source: PLoS One. 2017 Nov 2;12(11):e0187253. doi: 10.1371/journal.pone.0187253 (PMC5667819; doi:10.1371/journal.pone.0187253)
Supplement: S6 Appendix — (PDF) [file pone.0187253.s015.pdf]

Comparison between minimum, average and maximum height

Tables 1 and 2 show the dependence on, respectively, average and maximum height of all observables. Figures 1 and 2 provide, on the other hand, a graphical comparison for respectively the V and x observables. We show these two figures since these observables are mostly growing with height, and so their analysis is easier. As the figures show, the "average" curves are somehow in between the other two curves, with the "minimum" curve on the top and the maximum on the bottom, as expected for an observable that grows with height[1]. This suggests that dyads have, at least regarding height, a behaviour that is an average of the individual ones, and all three indicators should be basically equivalent. In the main text we choose to use the "minimum" height indicator because it allows to better identify dyads with children.

Table 1: Observable dependence on average height for dyads. Lengths in millimetres, times in seconds.

Average height	N_g^k	V	r	x	y
< 140 cm	14	1044 ± 52 (σ=195)	983 ± 98 (σ=365)	527 ± 39 (σ=145)	672 ± 130 (σ=492)
140-150 cm	22	1011 ± 36 (σ=168)	910 ± 81 (σ=382)	562 ± 39 (σ=183)	570 ± 100 (σ=476)
150-160 cm	118	1110 ± 23 (σ=253)	812 ± 24 (σ=260)	629 ± 14 (σ=149)	379 ± 31 (σ=340)
160-170 cm	472	1140 ± 8.3 (σ=181)	821 ± 12 (σ=250)	646 ± 7.6 (σ=165)	372 ± 15 (σ=329)
170-180 cm	421	1222 ± 8.7 (σ=179)	828 ± 11 (σ=224)	685 ± 8 (σ=164)	342 ± 14 (σ=282)
> 180 cm	42	1275 ± 27 (σ=174)	793 ± 26 (σ=171)	693 ± 19 (σ=122)	274 ± 34 (σ=220)
$F_{5,1083}$		17.9	1.95	7.31	5.74
p		$< 10^{-8}$	0.0829	$9.42 \cdot 10^{-7}$	$3.1 \cdot 10^{-5}$
R^2		0.0764	0.00894	0.0327	0.0258
δ		1.54	0.816	1.3	1.29

Table 2: Observable dependence on maximum height for dyads. Lengths in millimetres, times in seconds.

Maximum height	N_g^k	V	r	x	y
< 140 cm	3	1172 ± 12 (σ=21.4)	650 ± 49 (σ=84.4)	597 ± 23 (σ=39.1)	189 ± 60 (σ=104)
140-150 cm	3	1051 ± 79 (σ=136)	611 ± 11 (σ=19.6)	518 ± 39 (σ=67.5)	242 ± 33 (σ=57.2)
150-160 cm	49	988 ± 25 (σ=178)	782 ± 34 (σ=240)	594 ± 20 (σ=138)	366 ± 49 (σ=346)
160-170 cm	336	1129 ± 10 (σ=191)	820 ± 14 (σ=256)	637 ± 8.3 (σ=151)	378 ± 19 (σ=343)
170-180 cm	556	1191 ± 8.1 (σ=191)	830 ± 10 (σ=237)	671 ± 7.1 (σ=167)	359 ± 13 (σ=306)
> 180 cm	142	1250 ± 15 (σ=183)	843 ± 21 (σ=252)	677 ± 15 (σ=182)	365 ± 26 (σ=313)
$F_{5,1083}$		18.8	1.31	4.21	0.427
p		$< 10^{-8}$	0.257	0.000849	0.83
R^2		0.08	0.00601	0.0191	0.00197
δ		1.44	0.929	0.881	0.555

[1] A group whose tallest person is in, e.g, the 160-170 cm range is expected to have a shorter average height than a group whose shortest person is in the same range.

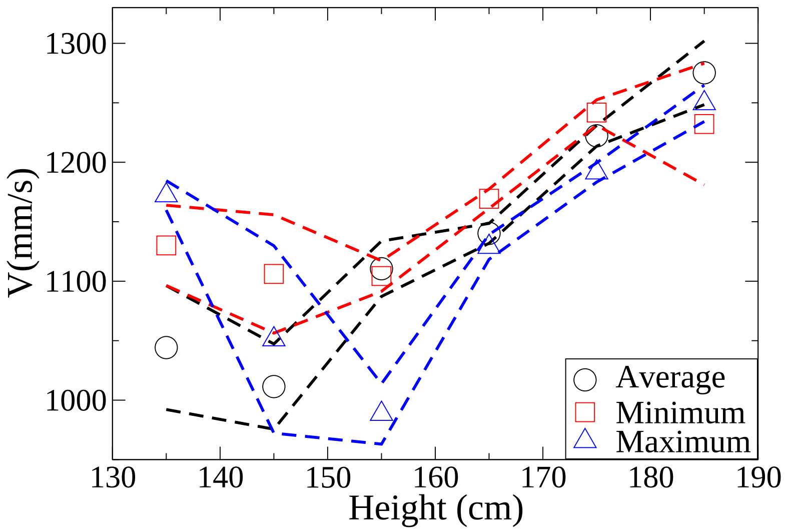

Figure 1: V dependence on average (black and circles), minimum (red and squares) and maximum (blue and triangles) height. Dashed lines provide standard error confidence bars. The points at 135 and 185 cm correspond to the “less than 140” and “more than 180” cm slots.

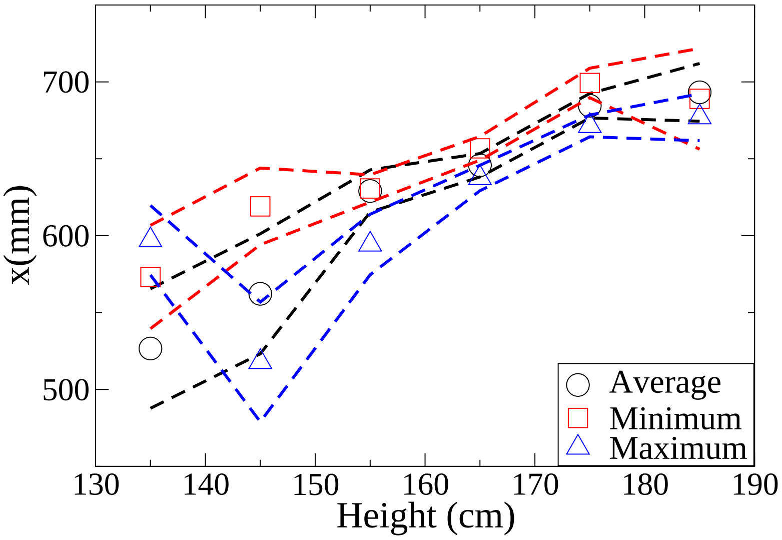

Figure 2: x dependence on average (black and circles), minimum (red and squares) and maximum (blue and triangles) height. Dashed lines provide standard error confidence bars. The points at 135 and 185 cm correspond to the “less than 140” and “more than 180” cm slots.
